# Resveratrol Alleviates Skeletal Muscle Insulin Resistance by Downregulating Long Noncoding RNA

**DOI:** 10.1155/2022/2539519

**Published:** 2022-01-19

**Authors:** Zhihong Liu, Zhimei Zhang, Guangyao Song, Xing Wang, Hanying Xing, Chao Wang

**Affiliations:** ^1^Department of Internal Medicine, Hebei Medical University, Shijiazhuang 050017, China; ^2^Endocrinology Department, Hebei General Hospital, Shijiazhuang 050051, China; ^3^Endocrinology Department, The Second Hospital of Hebei Medical University, Shijiazhuang 050000, China; ^4^Hebei Key Laboratory of Metabolic Diseases, Hebei General Hospital, Shijiazhuang 050051, China

## Abstract

Long noncoding RNA (lncRNA) is a crucial factor in the progression of insulin resistance (IR). Resveratrol (RSV) exhibits promising therapeutic potential for IR. However, there are few studies on whether RSV improves IR through lncRNA. This study aimed to determine whether RSV could influence the expression of lncRNA and to elucidate the underlying mechanism. Mice were divided into three groups: control group, high-fat diet (HFD) group, and HFD + RSV group. We conducted a high-throughput sequencing analysis to detect lncRNA and mRNA expression signatures and the ceRNA-network in the skeletal muscles of mice that were fed an HFD to induce IR. Hierarchical clustering, gene enrichment, and gene ceRNA-network analyses were subsequently conducted. Differentially expressed lncRNAs were selected and validated via reverse transcription-quantitative polymerase chain reaction (RT-qPCR). The biological functions of the selected lncRNAs were investigated by silencing the target genes via lentivirus transfection of C2C12 mouse myotube cells. RSV treatment reversed the expression of 338 mRNAs and 629 lncRNAs in the skeletal muscles of mice with HFD-induced IR. The results of the Gene Ontology and Kyoto Encyclopedia of Genes and Genomes database analyses indicated that the differentially expressed mRNAs modulated type II diabetes mellitus. After validating randomly selected lncRNAs via RT-qPCR, we identified a novel lncRNA, NONMMUT044897.2, which was upregulated in the HFD group and reversed with RSV treatment. Additionally, NONMMUT044897.2 was proven to function as a ceRNA of microRNA- (miR-) 7051-5p. Suppressor of Cytokine Signaling 1 (SOCS1) was confirmed as a target of miR-7051-5p. We further performed lentivirus transfection to knock down NONMMUT044897.2 *in vitro* and found that NONMMUT044897.2 silenced SOCS1 and potentiated the insulin signaling pathway. Hence, RSV mimicked the silencing effect of lentivirus transfection on NONMMUT044897.2. Our study revealed that RSV reduced IR in mouse skeletal muscles via the regulation of NONMMUT044897.2.

## 1. Introduction

Type 2 diabetes mellitus (T2DM) accounts for 90% of diabetes worldwide, and insulin resistance (IR) is a primary determinant of T2DM since it reduces glucose uptake and utilization [1]. Skeletal muscles play a significant role in the etiology of IR [2]. Phosphatidylinositol-3-kinase (PI3K)/protein kinase B (AKT) is the most important signaling pathway of insulin in glucose metabolism [3]. Activated AKT can promote phosphorylated GSK3*β*, inhibiting its activity to improve IR [4]. In recent years, researchers found that the Suppressor of Cytokine Signaling 1 (SOCS1) is an important negative regulator of the PI3K/AKT pathway [5, 6]. Overexpression of SOCS1 in the liver was found to reduce insulin sensitivity by downregulating the level of tyrosine phosphorylation of insulin receptor substrate in IR mice [5]. SOCS1 primarily inhibits the catalytic binding of insulin receptors to insulin receptor substrates to induce IR [6].

Resveratrol (3,5,4-trihydroxystilbene; RSV) is a natural polyphenol that is enriched in more than 70 kinds of plants [7]. Accumulating evidence has indicated that RSV has diverse biological activities [8, 9]. It functions as an antioxidant, antiaging, anti-inflammatory, hypoglycemic, and anti-IR agent [10, 11]. Several studies have reported that RSV possesses a significant anti-IR activity in skeletal muscles [12–14]. Our earlier research has also proven that skeletal muscle IR that is caused by an HFD may be alleviated with RSV treatment [14]. RSV increases the expression of microencapsulated protein 3 (CAV-3), thereby allowing skeletal muscle cells to carry glucose when the protein GLUT4 activates the transfer from the cytoplasm to the cell membrane, which in turn increases the ability of myocytes to transport glucose and improve IR [15]. Meanwhile, RSV attenuates insulin-stimulated AKT phosphorylation by eliminating insulin-induced ROS production in skeletal muscles [16].

Long noncoding RNAs (lncRNAs), a class of RNA molecules that are more than 200 nucleotides (nt) in length, have little or no protein-coding capacity [17]. Research has shown that lncRNAs widely participate in various developmental and physiological processes [18, 19]. In addition, they are strongly correlated with the development and progression of diseases, including coronary artery diseases [20], cancers [21], and metabolic diseases [22, 23]. Recently, lncRNAs have been confirmed to inhibit miRNA activity, increase miRNA target genes, and act as competitive endogenous RNAs (ceRNA) of miRNAs [24]. Further, the effect of lncRNAs in IR has been the focus of several studies [25, 26]. Previous studies [27, 28] by our group have demonstrated that RSV can improve hepatic IR by regulating lncRNA NONMMUT058999.2 and NONMMUT008655.2 in mouse models. However, the effect of RSV on IR through the regulation of the expression of lncRNAs in skeletal muscle remains unclear. In this study, we aimed to establish *in vivo* and *in vitro* IR models in skeletal muscles, which are a different tissue than those used in previous studies, to explore whether RSV can improve skeletal muscle IR by regulating lncRNAs.

## 2. Materials and Methods

### 2.1. Animal Experiments

We equally divided 42 healthy 6-week-old C57BL/6J background mice into three groups (*n* = 14 in each group): the control group, the HFD group, and the HFD + RSV group. The mice weighing around 22 g were purchased from the Beijing Viton Lihua Experimental Center (China) and sustained on a standard 12 h light-dark cycle, at 20–25°C and at 40–60% humidity. HFD mice were fed D12492J (20% protein, 20% carbohydrate, and 60% fat) for 8 weeks. The feed was purchased from Beijing Huafukang Biotechnology Co., Ltd. HFD + RSV mice were intragastrically fed 100 mg/kg/day RSV solution for 6 weeks following the methods outlined in our previous study [27]. Dissolved RSV (Sigma Aldrich, St. Louis, MO, USA) with dimethyl sulfolane (Sigma Aldrich; 30 mg mL^−1^) was diluted with 0.9% NaCl in a ratio of 1 : 2. The control group was fed D12450J (20% protein, 70% carbohydrate, and 10% fat). Weight and food intake were measured weekly during feeding. After the feeding experiment, all mice fasting for 12 h were intraperitoneally injected with 50% glucose (1.5 g kg^−1^ bodyweight) to conduct the glucose tolerance tests (intraperitoneal glucose tolerance test). Blood glucose was detected on the tail vein with a glucose meter at 0, 15, 30, 60, and 120 min after injection. The IR model was validated by determining the area under the curve in accordance with the protocol presented in our previously published study [29]. Animal studies (2019E369) were approved by the ethics committee of the Hebei General Hospital, and all animal experimental procedures complied with the National Institutes of Health guide for the care and use of laboratory animals.

### 2.2. Serum and Tissue Samples

Six mice in each group were randomly selected and intraperitoneally injected with 1.5 U/40 g of insulin (Sigma Aldrich). All mice in three groups were anesthetized by intraperitoneal injection of 2% sodium pentobarbital after 20 min. Blood samples were gathered via cardiac puncture and centrifuged at 3000 × *g* at 4°C for 10 min. The serum was then stored at −80°C for serological indicators. Collected skeletal muscles were withdrawn quickly and stored in liquid nitrogen for the follow-up study.

### 2.3. Serological Indicators

Detection kits for total cholesterol, triglyceride, high-density lipoprotein cholesterol, low-density lipoprotein cholesterol, and free fatty acids were acquired from Nanjing Jiancheng Institute of Biological Engineering (Jiangsu, China). Serum insulin was obtained using an ELISA kit (ALPCO Diagnostics, Salem, NH, USA). The manufacturer's protocol was followed in all the aforementioned procedures involving the detection kits.

### 2.4. Western Blot

The same amount of protein with different groups was separated by sodium dodecyl sulfate-polyacrylamide gel electrophoresis, transferred to polyvinylidene fluoride (PVDF) membrane (cat. no. ISEQ00010; Merk Millipore, Billerica, MA, USA), and sealed with 5% skimmed milk for 2 h. The primary antibodies were diluted in the blocking solution at the following concentrations: *β*-actin (cat. no. 4970; Cell Signaling Technology, Danvers, MA, USA): rabbit antibody, 1 : 5000; GAPDH (cat. no. 10494-1-AP; Proteintech Group, Inc. 5400 Pearl Street, Suite 300 Rosemont, IL 60018, USA): rabbit antibody, 1 : 10000; AKT (cat. no. 9272; Cell Signaling Technology): rabbit antibody, 1 : 1000; p-AKT (Ser 473) (cat. no. 9271; Cell Signaling Technology): rabbit antibody, 1 : 750; GSK3*β* (cat. no. 12456; Cell Signaling Technology): rabbit antibody, 1 : 750; p-GSK3*β* (cat. no. 5558; Cell Signaling Technology): rabbit antibody, 1 : 750; GLUT4 (cat. no. 2213; Cell Signaling Technology): mouse antibody, 1 : 5000; SOCS1 (cat. no. 3950; Cell Signaling Technology): rabbit antibody, 1 : 5000. PVDF membranes and primary antibodies were incubated at 4°C for 24 h. The membrane was then treated with secondary antibodies, stored at 30°C for about 50 min and washed three times for 10 min each time. The washed membrane was fully immersed in the iPer ECL Western HRP Substrate (cat. no. MF074-01; Mei5, Beijing, China) for about 2 min and exposed using the Gel Imager System (GDS8000; UVP, California, USA) to obtain the image of the target band. Protein bands were calculated by densitometry using the ImageJ software and were normalized to *β*-actin or GAPDH levels.

### 2.5. Reverse Transcription-Quantitative Polymerase Chain Reaction (RT-qPCR)

Total RNA was extracted using the Trizol reagent (TIANGEN, Beijing, China) from mouse skeletal muscle tissues and was reverse-transcribed into cDNA using HiScript II Q RT SuperMix for qPCR. RNA was tested for purity and concentration using NanoDrop 2000 (Thermo Fisher Scientific, Wilmington, DE, USA). Amplification was performed using the SYBr® Premix ex Taq II kit (RR820A; Takara Bio, Tokyo, Japan). The Applied Biosystems 7500 real-time PCR system was used to perform RT-qPCR, with a total of 41 cycles, including 3 minutes of predenaturation at 95°C, 5 seconds at 95°C, and 32 seconds at 60°C. The melting point curve was established at 60–95°C. *β*-Actin and U6 were considered as internal reference controls for genes. The relative gene expression was quantified by the 2^−(ΔΔCt)^ method [30]. The specific primers involved in this research are listed in Table 1.

### 2.6. cDNA Library Construction and RNA Sequencing

Total RNA was extracted using the RNeasy mini kit (Qiagen, Hilden, Germany) from skeletal muscle samples of four mice in each group. The TruSeq™ RNA Sample Preparation Kit (Illumina, San Diego, CA, USA) was used to make paired-end libraries following the manufacturer's instructions. Ribosomal RNA was removed using the Ribo-Zero rRNA Removal Kit (Epicentre, Madison, Wisconsin, USA), and then the mRNA was fragmented into small pieces with divalent cations. First chain cDNA was synthesized using reverse transcriptase and random primers, and second chain cDNA was generated by DNA Polymerase I and RNase H. Base “A” was added at the end of the cDNAs. Purified products were PCR-amplified to create the final cDNA library. Insert sizes were confirmed using a Qubit® 2.0 Fluorometer (Life Technologies, Carlsbad, CA, USA), and mole concentrations were calculated. Clusters were created using cBot and then sequenced on the Illumina NovaSeq 6000 (Illumina) by Sinotech Genomics Co., Ltd. (Shanghai, China). Each gene fragment was counted using the Stringtie software (version: 1.3.0; Johns Hopkins University, Baltimore, MD, USA) contrast and then normalized using the TMM (trimmed mean of M values) algorithm. The Fragments Per Kilobase Million value of each gene was then calculated. The high-throughput sequencing results were uploaded to the gene expression omnibus database (accession no. GSE178415).

## 3. Analysis of Differentially Expressed lncRNA and mRNA

The differential expression of skeletal muscle in the three groups was analyzed based on the “edge” package in R. The threshold of the *P*-value was confirmed by controlling the False Discovery Rate. The screening criteria for differential expression of mRNAs and lncRNAs were *P* < 0.05 and absolute value of log2FC >1.0.

### 3.1. Functional Group Analysis

The Gene Ontology (GO) and the Kyoto Encyclopedia of Genes and Genomes (KEGG) pathways determined the potential role of the lncRNAs that were coexpressed with the differentially expressed mRNAs. The GO analysis was conducted to establish significant annotations of genes and gene products in diversified organisms using the DAVID database (https://david.abcc.ncifcrf.gov). In addition, the KEGG pathway analysis was used to identify differentially expressed mRNAs in enriched pathways. *P* < 0.05 was identified as the threshold of significance.

### 3.2. Construction of a ceRNA Regulatory Network

Cytoscape (version 3.8.2) is a network visualization software with multiple applications for network analysis. It can be downloaded for free from https://www.cytoscape.org/.

### 3.3. C2C12 Cell Culture and Treatments

C2C12 mouse myotube cells were maintained in Dulbecco's modified Eagle medium (Gibco, Waltham, MA, USA) at a density of 5 × 10^4^ cells/cm^2^ containing 10% fetal bovine serum (San Diego, California, USA) and 1% penicillin/streptomycin (Wisent, Nanjing, China) at 37°C with 5% CO_2_. Cell differentiation was induced by incubation in Dulbecco's modified Eagle medium containing 2% fetal bovine serum for 4 days after reaching 80% confluence. Differentiated C2C12 cells at a density of 1 × 10^4^ cells/cm^2^ were incubated for 24 h with 0.25 mM palmitate (PA) (Aladdin Industries, China) [31]. At 0, 8, 16, and 24 h after the intervention of PA, the glucose concentration in the culture medium was measured by the glucose oxidase assay to determine whether IR in mice was established. Subcultured C2C12 cells were digested to prepare a cell suspension and subcultured to a 96-well culture plate. When the cells grew to about 80% confluence, RSV at concentrations of 100 *μ*M, 50 *μ*M, and 30 *μ*M was added to the medium. After 24 h, 10 *μ*L of CCK-8 was added to each well, which was protected against light. They were cultured for 20 min, and their absorbance was measured at 450 nm. The cell survival rate was then calculated. C2C12 cells (5 × 10^5^ cells/cm^2^) in the logarithmic growth phase were subcultured in a 6-well plate and transfected with lentivirus. Synthesized constructs included LV3-NC (5′ to 3′ TTCTCCGAACGTGTCACGT) and LV3-NONMMUT044897.2 (5′ to 3′ GCTCTTTCAGATAAGCCTTGT), which were obtained from GenePharma Co., Ltd. China. Stable cell lines were obtained after puromycin (2 *μ*g mL^−1^) selection for the PA-induced IR model and drug intervention experiments. The plated cells were grouped: the control group (CON), the PA group (PA), the PA + shRNA-NONMMUT044897.2 negative control group (PA + shRNA-NC), the PA + shRNA-NONMMUT044897.2 knockdown group (PA + shRNA-NONMMUT044897.2), and the PA + RSV 30 *μ*M group (PA + RSV). After the IR model was established, the glucose concentration was measured for 24 h, while the NONMMUT044897.2 and miR7051-5p mRNA levels were measured via RT-qPCR. The cells at a density of 5 × 10^5^ cells/cm^2^ were stimulated with 100 nM insulin, while the protein was extracted 20 min after insulin stimulation for the western blot analyses, and cells were extracted using the Trizol reagent (TIANGEN, Beijing, China).

### 3.4. Statistical Analyses

SPSS v23.0 was used for data analysis. The results are presented as a mean ± SD. Two-sample comparisons were analyzed using an independent sample *t*-test (Student's *t*-test). One-way ANOVA was used for statistical analysis followed by Bonferroni's multiple comparison test or Tamhane's multiple comparison test. *P* < 0.05 was regarded as statistically significant.

## 4. Results

### 4.1. RSV Ameliorates Body Weight, IR, and Lipid Levels in HFD-Fed Mice

After 6 weeks of RSV administration, body weight, fasting blood glucose, and insulin levels of the HFD + RSV group were greatly reduced compared with those of the HFD group (Table 2), although the daily caloric intake of the two groups was similar. The quantitative insulin sensitivity index of the HFD group was reduced compared with the indices of the CON and HFD + RSV groups (Table 2). RSV treatment reduced the upregulation of triglyceride, low-density lipoprotein cholesterol, and free fatty acids in the HFD group, while the total cholesterol was decreased but had no significance (Table 2). There was no difference in the high-density lipoprotein cholesterol of the mice (Table 2).

### 4.2. RSV Treatment Decreased SOCS1 and Increased the Phosphorylation of AKT and GSK3*β* in the HFD Group

**Figure 1:**
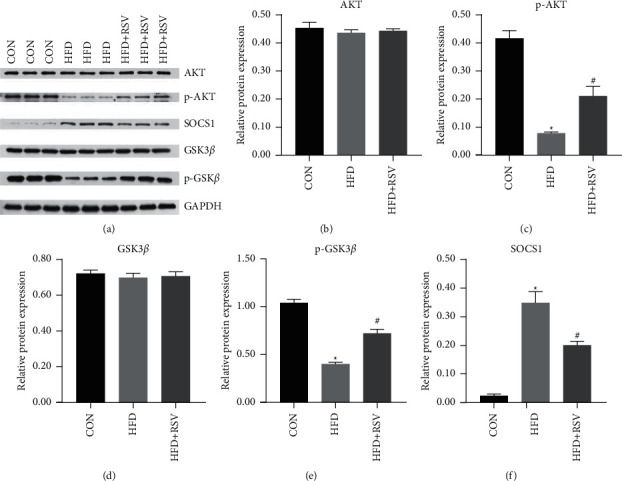
Effects of resveratrol on the insulin signaling pathway in control, HFD, and HFD + RSV groups. (a) Bands of western blot; (b) AKT; (c) p-AKT; (d) GSK3*β*; (e) p-GSK3*β*; (f) SOCS1. Data are expressed as the mean ± SD (*n* = 6). ^*∗*^*P* < 0.05 versus CON; ^#^*P* < 0.05 versus HFD.

Between the control, HFD, and HFD + RSV groups, no differences were found in the protein levels of AKT and GSK3*β* (Figures 1(a), 1(b), and 1(d)). The HFD group showed dramatic repression of p-AKT and p-GSK3*β* protein levels compared with those in the control group, while the RSV group showed a marked increase in p-AKT and p-GSK3*β* protein expression (Figures 1(a), 1(c), and 1(e)). Moreover, SOCS1 expression was abnormally elevated in the HFD group but decreased following RSV treatment (Figures 1(a) and 1(f)). These results suggest that RSV improves the expression of genes on the insulin signaling pathway.

### 4.3. RSV Systematically Modulates Skeletal Muscle Gene Expression

**Figure 2:**
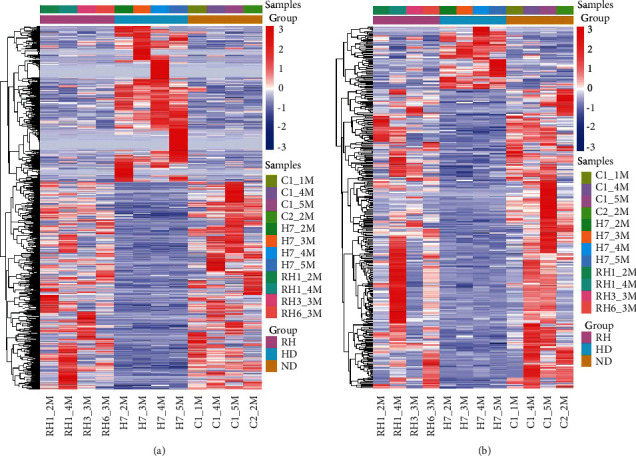
Profiles of differentially expressed genes in the three groups. Hierarchical clustering of lncRNAs (a) and mRNAs (b). Upregulated and downregulated lncRNAs (or mRNAs) are indicated in red and blue.

After standardization, 58,245 lncRNAs and 83,089 mRNAs were identified in the skeletal muscles of mice. On comparing the HFD group with the control group, we found that there were 3,276 differentially expressed lncRNAs (1,192 upregulated and 2,084 downregulated) and 2,118 differentially expressed mRNAs (314 upregulated and 1804 downregulated). Simultaneously, there were 1,640 differentially expressed lncRNAs (921 upregulated and 719 downregulated) and 604 differentially expressed mRNAs (444 upregulated and 160 downregulated) in the HFD + RSV group compared with those in the HFD group. Of the upregulated lncRNAs and mRNAs in the HFD group, 270 lncRNAs and 58 mRNAs were downregulated in the HFD + RSV group. Of the downregulated lncRNAs and mRNAs in the HFD group, 359 lncRNAs and 280 mRNAs were upregulated in the HFD + RSV group (Figures 2(a) and 2(b)). The top 30 differentially expressed lncRNAs and mRNAs are listed in Tables 3 and 4, with Fragments Per Kilobase Million = 0 eliminated. All differentially expressed lncRNAs and mRNAs in the three groups are listed in Supplementary Tables S1 and S2.

### 4.4. Functional Enrichment Analysis of Differentially Expressed Genes

**Figure 3:**
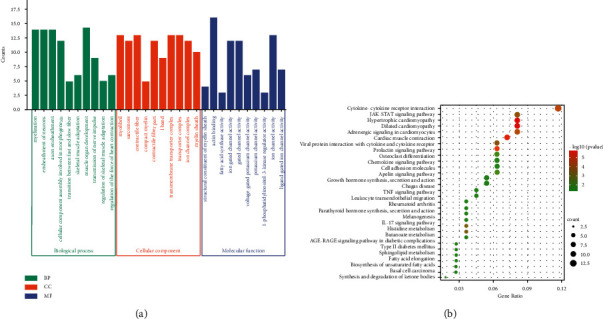
Gene Ontology (GO) and Kyoto Encyclopedia of Genes and Genomes (KEGG) pathway analysis of differentially expressed genes in the three groups. (a) Top 30 GO terms related to differentially expressed mRNAs. (b) Top 30 KEGG pathways related to differentially expressed mRNAs.

The functions of these different mRNAs were studied via enrichment analysis. The GO analysis was conducted to classify differentially expressed mRNAs into three types: the biological process, the molecular function, and the cellular component. The most highly enriched GO terms were “myelination, ensheathment of neurons, axon ensheathment, cellular component assembly involved in morphogenesis, transition between fast and slow fiber (biological process),” “myofibril, sarcomere, contractile fiber, compact myelin, contractile fiber part (cellular component),” and “structural constituent of myelin sheath, actin binding, fatty acid synthase activity, ion gated channel activity, and gated channel activity (molecular function)” (Figure 3(a)). KEGG analysis revealed that the differentially expressed mRNAs were mostly involved in cytokine-cytokine receptor interaction, the JAK-STAT signaling pathway, hypertrophic cardiomyopathy, the prolactin signaling pathway, and type II diabetes mellitus (Figure 3(b)).

### 4.5. RT-qPCR Validation *In Vivo*

**Figure 4:**
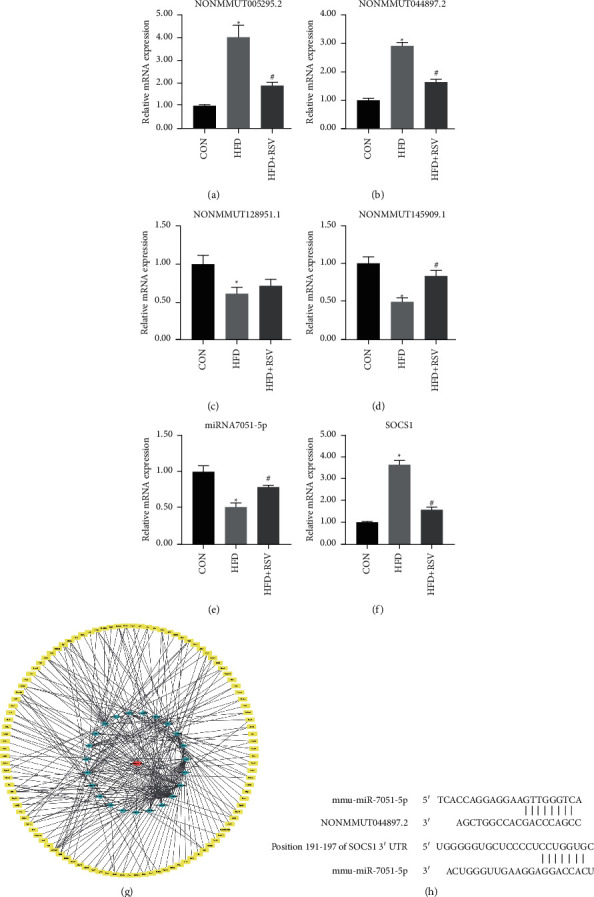
Validation of lncRNAs by RT-qPCR *in vivo*. Expression of NONMMUT005295.2 (a), NONMMUT044897.2 (b), NONMMUT128951.1 (c), NONMMUT145909.1 (d), miRNA-7051-5p mRNA (e), and SOCS1 mRNA (f) in different groups. (g) The NONMMUT044897.2 lncRNA-miRNA-mRNA network. (h) The positions of miR-7051-5p binding sites on NONMMUT044897.2 and the positions of miR-7051-5p binding sites on SOCS1. Data are expressed as the mean ± SD (*n* = 6). ^*∗*^*P* < 0.05 versus CON; ^#^*P* < 0.05 versus HFD.

To confirm the validity of the sequencing results, we randomly picked four differentially expressed lncRNAs, two upregulated lncRNAs in HFD, a downregulated HFD + RSV (NONMMUT044897.2; NONMMUT005295.2), and two lncRNAs with a reverse trend (NONMMUT128951.1; NONMMUT145909.1). The expression levels of the selected lncRNAs were consistent with those of the sequencing results (Figures 4(a)–4(d)), but there was no statistical difference in the increase of NONMMUT128951.1 in the HFD + RSV group. The SOCS1 mRNA level was increased in the HFD group and decreased with RSV treatment (Figure 4(f)). Of the verified lncRNAs, NONMMUT044897.2 had a higher expression level. In addition, the KEGG analysis indicated that SOCS1 played a vital role in the JAK-STAT signaling pathway and in type II diabetes mellitus. In the latter, SOCS1 was reportedly involved in the development of IR [5, 6]. To elucidate the interaction between NONMMUT044897.2 and SOCS1, we constructed a related lncRNA-miRNA-mRNA network diagram. The results revealed that NONMMUT044897.2 regulated SOCS1 through miR-7051-5p and miR-762 (Figure 4(g)). To further explore its potential molecular mechanism, we applied the NonCode and miRBase database analyses and found that there was base pairing in the sequence of NONMMUT044897.2 and miR-7051-5p. At the same time, according to the prediction results of TargetScan, SOCS1 was identified to be a miR-7051-5p target (Figure 4(h)). According to the above results, NONMMUT044897.2 might have regulated SOCS1 through miR-7051-5p. Therefore, this study further verified the expression of miR-7051-5p mRNA through RT-qPCR; miR-7051-5p mRNA was downregulated in the HFD group and upregulated in the HFD + RSV group (Figure 4(e)).

### 4.6. Establishment of a Cell Model of PA-Induced IR

**Figure 5:**
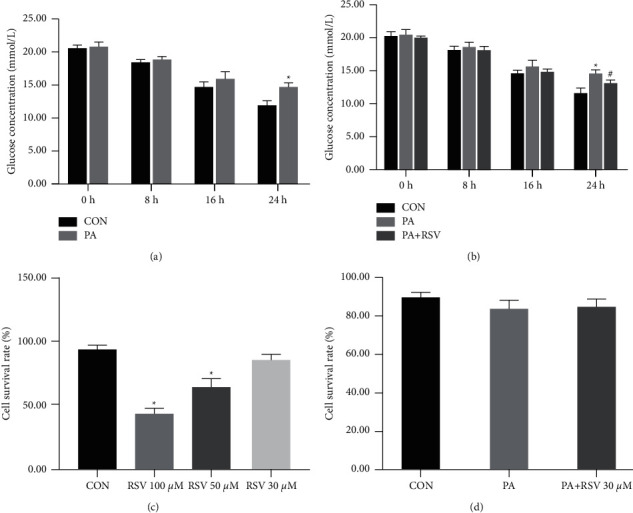
Resveratrol reduced PA-induced glucose concentration *in vitro*. Glucose concentration in the medium after 0, 8, 16, and 24 h treatment with PA (a) and treatment with PA and RSV (b). Cell survival rate after 24 h treatment with different concentrations of RSV (c) or PA and RSV treatments (d). Data are shown as the mean ± SD (*n* = 6). ^*∗*^*P* < 0.05 versus CON; ^#^*P* < 0.05 versus PA.

The C2C12 mouse myotube cells were transferred to media with and without 0.25 mM PA. Glucose concentrations were determined at 0, 8, 16, and 24 h. There was no significant difference in control and PA groups at 0, 8, and 16 h; however, the glucose concentration of the PA group was distinctly elevated at 24 h compared with that of the control group, thereby indicating that the IR model was successfully established (Figure 5(a)). Besides, the glucose concentration was obviously decreased at 24 h in the RSV group (Figure 5(b)).

The cell survival rate of C2C12 cells 24 h after RSV administration was approximately 30–100 *μ*m. The results showed that 30 *μ*m of RSV had no significant influence on the survival rate of C2C12 cells (Figure 5(c)). The cell survival rate of the PA group (84%) was lower than that of the control (89.6%), while that of the 30 *μ*M PA + RSV group (85%) was higher than that of the PA group. However, there were no statistical differences between these three groups (Figure 5(d)).

### 4.7. RT-qPCR Validation *In Vitro*

**Figure 6:**
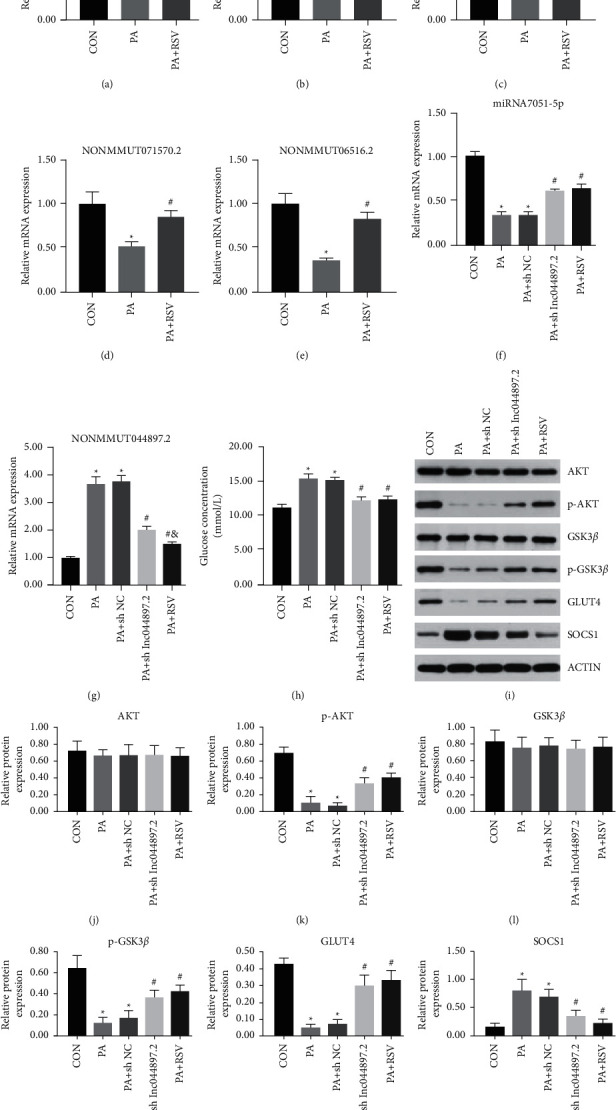
Resveratrol improved skeletal muscle insulin resistance by downregulating the lncRNA NONMUT044897.2 *in vitro*. Validation of lncRNAs NONMMUT044897.2 (a), NONMMUT139818.1 (b), NONMMUT00000181045(c), NONMMUT071570.2 (d), and NONMMUT06516.2 (e) by RT-qPCR. Expression of miR-7051-5p mRNA (f) and NONMMUT044897.2 mRNA (g) after shRNA transfection into C2C12 cells in different groups. (h) Glucose concentrations in the culture medium after shRNA transfection into C2C12 cells in different groups. (i) Protein bands of insulin signaling pathway-related molecules. Densitometric analysis of (j) AKT, (k) p-AKT, (l) GSK3*β*, (m) p-GSK3*β*, (n) GLUT4, and (o) SOCS1. Data are presented as the mean ± SD (*n* = 6). ^*∗*^*P* < 0.05 versus CON, ^#^*P* < 0.05 versus PA, and ^*δ*^*P* < 0.05 versus PA + shRNA-NONMMUT044897.2.

Two lncRNAs were upregulated in PA in which downregulation of PA + RSV (NONMMUT044897.2; NONMMUT139818.1) and upregulation of three lncRNAs (NONMMUT071570.2; NONMMUT065156.2; NONMMUT00000181045) were observed. The expression levels of the selected lncRNAs were consistent with those of the sequencing results (Figure 6(a)–6(e)), but there was no statistical difference in the increase of NONMMUT00000181045 in the PA + RSV group. To verify the relationship between NONMMUT044897.2 and RSV *in vitro*, C2C12 cells were transfected with lentivirus. The results of this transfection showed that, compared with the control group, NONMMUT044897.2 expression was robustly increased in the PA and PA + shRNA-NC groups. The PA + shRNA-NONMMUT044897.2 group had a decreased expression of NONMMUT044897.2 compared with that of the PA group. RSV administration also resulted in a decrease in the expression of NONMMUT044897.2 compared with that of the PA group (Figure 6(g)).

Relative to the control group, miR-7051-5p mRNA expression in the PA and PA + shRNA-NC groups was significantly reduced, while knockdown of NONMMUT044897.2 and RSV treatment markedly increased the miR-7051-5p mRNA expression level (Figure 6(f)). The concentration of glucose in the media of the PA and PA + shRNA-NC groups was substantially increased compared with that of the control group. NONMMUT044897.2 silencing and RSV treatment strikingly overturned the glucose concentrations in the medium (Figure 6(h)). Knockdown of NONMMUT044897.2 distinctively upregulated the p-AKT, p-GSK3*β*, and GLUT4 protein levels and greatly reduced the SOCS1 protein level, compared with those of the PA group. RSV treatment had a similar effect in terms of NONMMUT044897.2 silencing on p-AKT, p-GSK3*β*, GLUT4, and SOCS1 protein levels (Figures 6(i)–6(o)).

## 5. Discussion

A large number of experiments have verified the therapeutic effect of RSV in IR [32, 33]. In this study, we demonstrated that RSV influenced the amelioration of IR in HFD-fed mice. These findings have also been reported by several studies [11, 12]. In the HFD + RSV group, blood glucose, insulin index, blood lipids, and area under the curve were decreased. RSV treatment ameliorated HFD-induced IR in mice by restoring the insulin signaling pathway gene expression. After intervention with RSV, the protein expression levels of p-AKT, p-GSK3*β*, and GLUT4 increased significantly.

High-throughput sequencing showed that there were 3,276 differentially expressed lncRNAs and 2,118 differentially expressed mRNAs in the HFD + RSV group, as compared with those in the control group, which yielded 1,640 differentially expressed lncRNAs and 604 differentially expressed mRNAs. We further found 338 mRNAs and 629 lncRNAs whose expression was reversed in the HFD and the HFD + RSV groups, suggesting that RSV plays a significant role in the overall alteration of skeletal muscle gene expression. Moreover, RT-qPCR results revealed that RSV ameliorated IR by regulating the expression of lncRNAs in skeletal muscles. As mentioned above, the verified lncRNAs were consistent with those of the sequencing results. In addition, NONMMUT044897.2 was highly expressed. The KEGG analysis uncovered that the differential genes were part of T2DM. We found that NONMMUT044897.2 was associated with SOCS1, which was critically involved in T2DM. Overexpression of SOCS1 aggravated IR [5], which played an important role in T2DM. Hence, we selected this lncRNA for further study, which has not been reported before.

SOCS1 is a specific negative regulator that regulates the JAK/STAT pathway [34]. The expression of SOCS1 increases with IR and decreases with the phosphorylation of IRS-1. Overexpression of SOCS-1 could inhibit insulin-induced glycogen synthesis in L6 myotubes [6]. AKT has essential roles in many signaling pathways, such as cell survival and cell metabolism. AKT is the center of the insulin signaling pathway, which regulates glucose and lipid metabolism. Activated AKT can stimulate the translocation of insulin-sensitive GLUT4 to the cell membrane through its downstream substrate ASl60 to increase glucose uptake. It can also phosphorylate GSK3*β* to inhibit its activity, promote glycogen synthesis, lower blood sugar, and improve IR [35]. Furthermore, an HFD could result in the decrease of skeletal muscle IRS-1, P13K, AKT, and GLUT4 gene expression levels and reduce p-AKT (ser473) and p-GSK3*β* protein expression levels. Studies have reported that overexpression of SOCS1 may inhibit the phosphorylation and activation of IRS-1 [6, 34], which in turn inhibits the activation of AKT, thereby indicating that there is an important link between AKT and SOCS1. We found that, in the IR model mice, mRNA and protein expression levels of SOCS1 were significantly increased, while RSV treatment exhibited the reverse trend, thereby improving IR and decreasing blood glucose levels.

Numerous studies have demonstrated that lncRNAs may be involved in human diseases by regulating the expression of miRNAs [36, 37]. The ceRNA-network diagram uncovered the possible regulatory roles of candidate lncRNAs. NONMMUT044897.2 could regulate SOCS1 through two different miRNAs. To further clarify the relationship between NONMMUT044897.2 and SOCS1, we constructed NONMMUT044897.2 and miR-7051-5p, miR-7051-5p, and SOCS1 base-pairing maps based on NonCode, miRBase, and TargetScan databases. These results indicate that NONMMUT044897.2 might have regulated the expression of SOCS1 through miR-7051-5p. Future studies should perform luciferase assays to verify the interactions between NONMMUT044897.2, miR-7051-5p, and SOCS1.

We confirmed that knockdown of NONMMUT044897.2 increased miR-7051-5p levels and promoted the expression of genes that are involved in the insulin signaling pathways (p-AKT, p-GSK3*β*, and GLUT4). Meanwhile, the expression of SOCS1 was suppressed by silencing NONMMUT044897.2. Moreover, knockdown of NONMMUT044897.2 has led to reduced glucose concentration, which is similar to the phenotypes induced with RSV treatment. This indicates that RSV improves skeletal muscle IR through downregulating the lncRNA NONMMUT044897.2.

## 6. Conclusions

This research profiled the differential expression of lncRNAs between the IR model mice and those treated with RSV. We further revealed the potentially regulated lncRNA NONMMUT044897.2. More work remains to be done to prove the relationship between the NONMMUT044897.2/miR-7051-5p/SOCS1 and RSV in the IR model. The study also has several limitations. First, *in vivo* animal models are needed to further silence NONMMUT044897.2 to verify the influence on IR. Second, the overexpression of NONMMUT044897.2 should be performed *in vitro*. Third, knockdown of the NONMMUT044897.2 in the RSV group should be done to observe its influence on the IR model. Overall, our data indicated that RSV could promote skeletal muscle IR, at least partially, via a lncRNA NONMMUT044897.2/miR-7051-5p/SOCS1 pathway. This provides a new perspective for the RSV treatment of IR in skeletal muscles.

## Figures and Tables

**Table 1:** Primers used for real-time quantitative polymerase chain reaction.

Gene	Forward primer (5′–3′)	Reverse primer (5′–3′)
*β*-Actin	GGCGCTTTTGACTCAGGATT	GGGATGTTTGCTCCAACCAA
NONMMUT139818.1	TGGGTCCTTGGTGTTCTTGTT	TCTAAAGTGGAGCCAACAAAGG
NONMMUT044897.2	TCCCAAAGAGTTCCGAAGGTA	GTGATGACACCAGGTATGACGG
NONMMUT005295.2	AGGCTTGTCTGAGGTTGCTGG	TTTACATCCTTGGGCTGCTTT
NONMMUT071570.2	TCTCCTGGGCTTCCCTAACTAA	CTCCCAAGGGCAGCATAACA
NONMMUT065156.2	GTTGCCATTCATCCTACCTCTTC	ATCAAATGAAAACCAACCCCG
NONMMUT00000181045	CAGCCAAATCACCAACAAACAGA	CCCTTACTCATAAATCAGCCTCACC
NONMMUT128951.1	GCTGGTCAAGCCAACAAGTAGT	GGCACCACATTGAACAGTAAAGTC
NONMMUT145909.1	AAGGGTGGACCAAGGCTAAAC	ACTGGCATCCTCAAACCTCAA
AKT	AAGGAGGTCATCGTCGCCAA	ACAGCCCGAAGTCCGTTATC
GSK3*β*	AAGGACTCACCAGGAGCAGGA	ATGTGGAGGGATAAGGATGGTG
SOCS1	CCGTGACTACCTGAGTTCCTTC	ATGAGGTCTCCAGCCAGAAGTG
mmu-miR-7051-5p	CTCAACTGGTGTCGTGGAGTCGGCAATTCAGTTGAGTGACCCAA	ACACTCCAGCTGGGTCACCAGGAGGAAGTT
U6	CTCGCTTCGGCAGCACA	AACGCTTCACGAATTTGCGT

**Table 2:** General indicators after resveratrol treatment.

Name	CON group (*n* = 14)	HFD group (*n* = 14)	HFD + RSV group (*n* = 14)
Initial body weight (g)	22.40 ± 1.22	22.95 ± 1.11	22.32 ± 1.14
Final body weight (g)	27.04 ± 2.56	41.61 ± 3.71^*∗*^	38.83 ± 2.27^#^
Food intake (kcal/d)	12.89 ± 0.15	12.50 ± 0.30	12.52 ± 0.72
TC (mmol/L)	6.06 ± 0.72	7.62 ± 0.52^*∗*^	7.44 ± 0.43
TG (mmol/L)	0.51 ± 0.11	1.42 ± 0.22^*∗*^	0.86 ± 0.07^#^
HDL-C (mmol/L)	3.26 ± 0.22	3.01 ± 0.30	3.14 ± 0.37
LDL-C (mmol/L)	0.21 ± 0.02	0.64 ± 0.07^*∗*^	0.47 ± 0.04^#^
FFA (mmol/L)	0.73 ± 0.08	1.22 ± 0.14^*∗*^	0.93 ± 0.05^#^
FBG (mmol/L)	5.9 ± 0.70	12.42 ± 1.96^*∗*^	8.14 ± 0.85^#^
Insulin (ng/mL)	0.20 ± 0.20	1.35 ± 0.28^*∗*^	0.51 ± 0.21^#^
QUICKI	0.83 ± 0.17	0.41 ± 0.02^*∗*^	0.55 ± 0.05^#^

^*∗*^ *P* < 0.05 versus CON; ^#^*P* < 0.05 versus HFD. TC: total cholesterol; TG: triglyceride; HDL-C: high-density lipoprotein cholesterol; LDL-C: low-density lipoprotein cholesterol; FFA: free fatty acid; FBG: fasting blood sugar; QUICKI: quantitative insulin sensitivity index; HFD: high-fat diet.

**Table 3:** Top 30 significantly differentially expressed lncRNAs in mice.

LncRNA_id	log2FC (HFD versus CON)	*P* value (HFD versus CON)	Up/down (HFD versus CON)	log2FC (HFD + RSV versus HFD)	*P* value (HFD + RSV versus HFD)	Up/down (HFD + RSV versus HFD)
NONMMUT147944.1	7.361654305	3.08*E*−05	Up	−7.65705	2.59*E*−05	Down
NONMMUT018494.2	6.726119826	2.72*E*−30	Up	−6.63171	1.25*E*−30	Down
NONMMUT056862.2	6.372532912	0.000101	Up	−6.47057	0.000104	Down
NONMMUT001029.2	5.951073546	1.73*E*−06	Up	−6.20954	1.09*E*−06	Down
NONMMUT034722.2	5.925450571	5.74*E*−05	Up	−5.99116	5.98*E*−05	Down
NONMMUT042491.2	5.439454581	0.000152	Up	−6.0331	6.81*E*−05	Down
NONMMUT011659.2	5.319133003	0.000118	Up	−5.39538	0.000122	Down
NONMMUT028972.2	5.109498067	9.40*E*−05	Up	−5.33988	7.69*E*−05	Down
NONMMUT056994.2	4.330643092	1.11*E*−07	Up	−3.97586	2.22*E*−06	Down
NONMMUT044528.2	4.3149951	2.45*E*−05	Up	−4.67614	1.12*E*−05	Down
NONMMUT139818.1	4.27510251	5.42*E*−10	Up	−2.93598	3.09*E*−07	Down
NONMMUT006490.2	4.040499067	4.37*E*−05	Up	−4.25073	3.25*E*−05	Down
NONMMUT153460.1	3.987910041	1.14*E*−10	Up	−4.43375	1.79*E*−11	Down
NONMMUT047957.2	3.622830463	5.31*E*−12	Up	−2.17202	1.80*E*−06	Down
NONMMUT061044.2	3.580172631	0.000913	Up	−5.02265	0.000116	Down
NONMMUT041793.2	3.348027654	0.000102	Up	−4.15666	2.47*E*−06	Down
MSTRG.26789.5	3.346146359	4.90*E*−17	Up	−2.35799	1.25*E*−05	Down
NONMMUT141647.1	3.045932847	2.40*E*−05	Up	−3.7519	3.30*E*−05	Down
NONMMUT019242.2	2.994018555	1.34*E*−16	Up	−1.86027	2.37*E*−07	Down
NONMMUT054892.2	2.304363454	8.76*E*−08	Up	−1.99848	3.44*E*−05	Down
NONMMUT051479.2	2.213054794	0.00098	Up	−3.05151	3.84*E*−05	Down
NONMMUT003238.2	2.187198497	0.045286	Up	−5.56027	8.43*E*−05	Down
NONMMUT004274.2	2.077962645	1.51*E*−05	Up	−1.86821	0.000128	Down
NONMMUT006717.2	2.041245592	1.41*E*−06	Up	−2.02194	8.66*E*−06	Down
ENSMUST00000145549	1.981088074	3.80*E*−07	Up	−1.97828	7.99*E*−07	Down
NONMMUT071342.2	1.630473739	0.012079	Up	−3.80688	1.60*E*−13	Down
NONMMUT005295.2	1.449638857	1.11*E*−06	Up	−1.41758	2.03*E*−05	Down
NONMMUT070926.2	1.34488241	0.026106	Up	−2.66561	8.38*E*−08	Down
NONMMUT006953.2	1.275954985	0.000623	Up	−2.36716	2.40*E*−09	Down
NONMMUT044897.2	1.249501171	2.60*E*−05	Up	−1.43116	2.19*E*−05	Down
NONMMUT048831.2	−1.272978476	0.046708	Down	1.547263	0.000408	Up
NONMMUT018620.2	−1.425732816	0.032203	Down	1.762165	0.000445	Up
NONMMUT051818.2	−1.49981749	0.00019	Down	1.757541	0.000229	Up
NONMMUT148959.1	−1.821849383	0.022286	Down	2.39635	0.000835	Up
NONMMUT025210.2	−1.865766716	0.032229	Down	3.501751	1.41*E*−09	Up
NONMMUT024340.2	−1.99805347	6.44*E*−07	Down	1.727535	0.000114	Up
NONMMUT030788.2	−2.072643561	0.008364	Down	3.386966	6.32*E*−08	Up
NONMMUT083064.1	−2.184391746	0.02567	Down	3.578567	9.44*E*−07	Up
NONMMUT117757.1	−2.220356685	0.000126	Down	1.946318	0.000268	Up
NONMMUT145026.1	−2.225432629	0.005502	Down	2.733938	0.00075	Up
NONMMUT143802.1	−2.239123899	0.000257	Down	2.484837	0.000105	Up
NONMMUT081465.1	−2.275670974	0.000759	Down	2.214893	0.000569	Up
NONMMUT071570.2	−2.342779878	1.52*E*−05	Down	2.040383	7.76*E*−05	Up
NONMMUT145721.1	−2.395813608	0.00904	Down	3.144452	0.000475	Up
NONMMUT082610.1	−2.721997741	0.004713	Down	3.500026	3.04*E*−06	Up
NONMMUT032162.2	−2.756781381	0.000975	Down	3.252807	1.74*E*−06	Up
NONMMUT098269.1	−2.860045031	2.20*E*−09	Down	2.306565	0.000783	Up
NONMMUT119847.1	−2.871001568	6.07*E*−06	Down	2.197726	0.000589	Up
NONMMUT004497.2	−3.006495667	6.12*E*−06	Down	3.18202	2.60*E*−06	Up
NONMMUT144862.1	−3.021747289	0.000339	Down	2.825872	0.000763	Up
NONMMUT152140.1	−3.390813528	0.005418	Down	3.671173	0.000159	Up
NONMMUT145717.1	−3.429960838	0.003878	Down	4.82329	1.67*E*−05	Up
ENSMUST00000181045	−3.5774189	2.18*E*−05	Down	4.111429	1.20*E*−07	Up
MSTRG.8668.1	−3.650059914	8.30*E*−13	Down	2.418835	0.0002	Up
NONMMUT145909.1	−4.44549442	6.50*E*−05	Down	3.575655	3.04*E*−05	Up
NONMMUT008421.2	−4.495334741	0.000753	Down	5.182219	4.44*E*−06	Up
ENSMUST00000161890	−5.096061065	0.013794	Down	6.206952	7.53*E*−05	Up
NONMMUT128951.1	−5.845021433	2.88*E*−07	Down	4.876355	2.89*E*−05	Up
NONMMUT065156.2	−7.177240343	5.91*E*−41	Down	6.653339	2.22*E*−10	Up
NONMMUT026869.2	−8.335434963	1.59*E*−11	Down	7.485915	1.33*E*−06	Up

Top 30 upregulated or downregulated lncRNAs in three groups. HFD: high-fat diet; CON: control; RSV: resveratrol; lncRNA: long noncoding RNA.

**Table 4:** Top 30 significantly differential expression mRNAs in mice.

Gene name	log2FC (HFD versus CON)	*P* value (HFD versus CON)	Up/down (HFD versus CON)	log2FC (HFD + RSV versus HFD)	*P* value (HFD + RSV versus HFD)	Up/down (HFD + RSV versus HFD)
Gm49388	6.06138	1.92*E*−10	Up	−2.53021	0.044372	Down
Gm26876	3.811306	7.03*E*−09	Up	−3.11381	5.15*E*−07	Down
4930512H18Rik	3.787461	3.47*E*−18	Up	−1.20868	0.003987	Down
Gm29676	3.283294	0.00530139	Up	−3.86133	0.002862	Down
Map6d1	3.074208	0.007729399	Up	−1.72651	0.047048	Down
Ppm1n	3.000682	2.93*E*−08	Up	−1.05825	0.012598	Down
Sh3gl2	2.803522	1.83*E*−09	Up	−2.08107	5.64*E*−06	Down
Clca4a	2.729295	0.000758257	Up	−2.14669	0.002907	Down
Gm49347	2.69398	0.016676861	Up	−2.79388	0.016914	Down
Gm33543	2.661545	2.23*E*−13	Up	−1.30568	0.029604	Down
Ccl12	2.641596	0.005800303	Up	−3.96508	0.00141	Down
Gm9402	2.633552	0.044305865	Up	−2.95627	0.046539	Down
Gm48719	2.618363	1.87*E*−05	Up	−1.43542	0.008835	Down
Rpl21-ps12	2.537707	0.000902295	Up	−1.83961	0.012602	Down
Gm15478	2.466703	1.04*E*−05	Up	−1.16718	0.029683	Down
Kcnh7	2.453351	3.86*E*−11	Up	−1.11192	0.016375	Down
Socs1	2.44948	4.05*E*−07	Up	−2.36143	1.61*E*−06	Down
Gm47603	2.39615	2.51*E*−05	Up	−1.36179	0.007302	Down
C130026L21Rik	2.31491	0.023709939	Up	−2.52323	0.024031	Down
AY036118	2.26368	0.014427254	Up	−2.08364	0.022577	Down
Cish	2.1587	1.43*E*−14	Up	−2.48736	1.64*E*−20	Down
Cd209e	2.146486	0.001095503	Up	−1.52077	0.033734	Down
Dkk3	2.060882	1.16*E*−07	Up	−1.51887	0.000588	Down
Pou2f3	2.046599	0.032113693	Up	−2.67227	0.010465	Down
Pcsk1	1.960889	6.87*E*−07	Up	−1.14143	0.001394	Down
Gm26635	1.857674	0.020348988	Up	−1.53523	0.040218	Down
B230312C02Rik	1.846494	1.97*E*−10	Up	−1.85503	2.38*E*−10	Down
4932438H23Rik	1.740581	0.019356615	Up	−1.8725	0.019401	Down
Ccl7	1.669165	0.006830603	Up	−1.92417	0.005174	Down
Megf11	1.597213	0.002069216	Up	−1.26422	0.028085	Down
Sox10	−1.00199	0.00499603	Down	1.277467	0.008898	Up
Gm17971	−1.01911	0.001132241	Down	1.082473	0.012236	Up
Mgat3	−1.02613	0.002140658	Down	1.038285	0.007315	Up
Gas2l3	−1.02807	0.020453198	Down	1.139046	0.006303	Up
Bcas1	−1.04182	0.015404068	Down	1.7179	0.002295	Up
Asphd2	−1.04528	0.020154215	Down	1.010603	0.025998	Up
Gm37537	−1.05529	0.004647624	Down	1.013631	0.014094	Up
Fos	−1.07258	0.000205959	Down	1.126982	0.002405	Up
Gldn	−1.07534	0.029747688	Down	1.119705	0.011748	Up
Pmp22	−1.10369	0.0243704	Down	1.272211	0.007779	Up
Sptbn5	−1.10708	0.002406818	Down	1.274	0.003898	Up
Plekha4	−1.11677	0.003070017	Down	1.306669	0.002928	Up
Kcna6	−1.11915	0.010919737	Down	1.15063	0.011805	Up
Gabrr1	−1.12133	0.007296347	Down	1.004214	0.027277	Up
Mir1904	−1.12175	0.004769582	Down	1.111331	0.011967	Up
Kif19a	−1.14672	0.006248502	Down	1.502258	0.001265	Up
Fosl2	−1.16247	1.85*E*−08	Down	1.02334	4.21*E*−05	Up
Lrrc71	−1.16508	0.023094219	Down	1.001178	0.048202	Up
Fhl4	−1.1723	0.034470511	Down	1.121767	0.045133	Up
Fa2h	−1.18303	0.034450796	Down	1.299243	0.029667	Up
Gm37510	−1.19184	0.004453269	Down	1.03012	0.008302	Up
Ppia	−1.19672	0.001831193	Down	1.119291	2.62*E*−05	Up
Tnfsf13b	−1.20579	0.016517031	Down	1.142204	0.010129	Up
Tox	−1.21833	0.015176461	Down	1.414292	0.001254	Up
Rasgef1c	−1.2224	0.036363327	Down	1.405989	0.013403	Up
Mt3	−1.22738	0.007211092	Down	1.291177	0.005362	Up
Elovl7	−1.24473	0.016851444	Down	1.412536	0.009919	Up
Wnt2b	−1.26686	0.007582771	Down	1.527884	0.008061	Up
Tenm2	−1.27034	0.005050296	Down	1.05799	0.010664	Up
Mmp27	−1.27782	0.027735632	Down	1.191382	0.04508	Up

The table lists the top 30 of the results for mRNA with upregulation or downregulation in expression in three groups. HFD: high-fat diet; CON: control; RSV: resveratrol; lncRNA: long noncoding RNA.

## Data Availability

The datasets used and/or analyzed during the current study are available from the corresponding author on reasonable request.

## Supplementary Materials

Supplementary Materials All differentially expressed lncRNAs and mRNAs in the control, HFD, and HFD + RSV groups are listed in Supplementary Tables S1 and S2.
