# Supplementary material for: Resveratrol Alleviates Skeletal Muscle Insulin Resistance by Downregulating Long Noncoding RNA
Source: Int J Endocrinol. 2022 Jan 19;2022:2539519. doi: 10.1155/2022/2539519 (PMC8791716; doi:10.1155/2022/2539519)
Supplement: Supplementary Materials — All differentially expressed lncRNAs and mRNAs in the control, HFD, and HFD + RSV groups are listed in Supplementary Tables S1 and S2. [file 2539519.f1.zip › 2539519.f1/Supplementary Table 1.pdf]

All_differentially_expressed_lncRNAs_in_the__control__HFD__and_HFD+RSV_groups

lncRNA_id	log2FC	P-value	updown (RHvs HD)	log2FC	P-value	updown (HD vs ND)
NONMMUT151772.1	Inf	1.46E-15	UP	#NAME?	6.23E-05	DOWN
NONMMUT044149.2	Inf	3.38E-14	UP	#NAME?	2.70E-05	DOWN
NONMMUT065156.2	6.653339	2.22E-10	UP	-7.17724	5.91E-41	DOWN
NONMMUT062734.2	Inf	2.40E-10	UP	#NAME?	0.000222	DOWN
ENSMUST00000144591	Inf	1.21E-09	UP	#NAME?	8.00E-06	DOWN
NONMMUT025210.2	3.501751	1.41E-09	UP	-1.86577	0.032229	DOWN
NONMMUT003237.2	Inf	5.24E-08	UP	#NAME?	0.000933	DOWN
NONMMUT030788.2	3.386966	6.32E-08	UP	-2.07264	0.008364	DOWN
NONMMUT066321.2	Inf	7.30E-08	UP	#NAME?	8.58E-05	DOWN
ENSMUST00000181045	4.111429	1.20E-07	UP	-3.57742	2.18E-05	DOWN
NONMMUT004498.2	Inf	3.46E-07	UP	#NAME?	4.77E-05	DOWN
NONMMUT083064.1	3.578567	9.44E-07	UP	-2.18439	0.02567	DOWN
NONMMUT026869.2	7.485915	1.33E-06	UP	-8.33543	1.59E-11	DOWN
NONMMUT032162.2	3.252807	1.74E-06	UP	-2.75678	0.000975	DOWN
NONMMUT004497.2	3.18202	2.60E-06	UP	-3.0065	6.12E-06	DOWN
NONMMUT082610.1	3.500026	3.04E-06	UP	-2.722	0.004713	DOWN
ENSMUST00000134845	Inf	3.64E-06	UP	#NAME?	0.000545	DOWN
NONMMUT008421.2	5.182219	4.44E-06	UP	-4.49533	0.000753	DOWN
NONMMUT141013.1	Inf	6.55E-06	UP	#NAME?	0.013846	DOWN
NONMMUT042593.2	Inf	6.80E-06	UP	#NAME?	0.000743	DOWN
NONMMUT095117.1	Inf	7.08E-06	UP	#NAME?	5.61E-05	DOWN
NONMMUT087867.1	Inf	1.18E-05	UP	#NAME?	0.000137	DOWN
NONMMUT152957.1	Inf	1.37E-05	UP	#NAME?	0.002989	DOWN
NONMMUT082499.1	Inf	1.46E-05	UP	#NAME?	0.001765	DOWN
ENSMUST00000182265	Inf	1.48E-05	UP	#NAME?	7.48E-06	DOWN
NONMMUT145717.1	4.82329	1.67E-05	UP	-3.42996	0.003878	DOWN
NONMMUT103354.1	Inf	2.27E-05	UP	#NAME?	3.05E-05	DOWN
NONMMUT128951.1	4.876355	2.89E-05	UP	-5.84502	2.88E-07	DOWN
NONMMUT145909.1	3.575655	3.04E-05	UP	-4.44549	6.50E-05	DOWN
ENSMUST00000142678	Inf	3.32E-05	UP	#NAME?	0.028613	DOWN
ENSMUST00000161890	6.206952	7.53E-05	UP	-5.09606	0.013794	DOWN
NONMMUT071570.2	2.040383	7.76E-05	UP	-2.34278	1.52E-05	DOWN
NONMMUT143802.1	2.484837	0.000105	UP	-2.23912	0.000257	DOWN
NONMMUT024340.2	1.727535	0.000114	UP	-1.99805	6.44E-07	DOWN
ENSMUST00000180835	Inf	0.000119	UP	#NAME?	0.037227	DOWN
NONMMUT152140.1	3.671173	0.000159	UP	-3.39081	0.005418	DOWN
NONMMUT041113.2	Inf	0.000166	UP	#NAME?	0.000485	DOWN
NONMMUT015425.2	Inf	0.000168	UP	#NAME?	2.28E-08	DOWN
MSTRG.8668.1	2.418835	0.0002	UP	-3.65006	8.30E-13	DOWN
NONMMUT051818.2	1.757541	0.000229	UP	-1.49982	0.00019	DOWN
NONMMUT098052.1	Inf	0.000256	UP	#NAME?	6.31E-05	DOWN
NONMMUT033805.2	Inf	0.000258	UP	#NAME?	4.09E-06	DOWN
NONMMUT117757.1	1.946318	0.000268	UP	-2.22036	0.000126	DOWN
NONMMUT039810.2	Inf	0.000315	UP	#NAME?	0.024073	DOWN
ENSMUST00000063376	Inf	0.000337	UP	#NAME?	0.000182	DOWN
ENSMUST00000181719	Inf	0.000342	UP	#NAME?	0.00085	DOWN

NONMMUT020965.2	Inf	0.00038	UP	#NAME?	0.024403	DOWN
NONMMUT048831.2	1.547263	0.000408	UP	-1.27298	0.046708	DOWN
NONMMUT018620.2	1.762165	0.000445	UP	-1.42573	0.032203	DOWN
NONMMUT145721.1	3.144452	0.000475	UP	-2.39581	0.00904	DOWN
NONMMUT081465.1	2.214893	0.000569	UP	-2.27567	0.000759	DOWN
NONMMUT119847.1	2.197726	0.000589	UP	-2.871	6.07E-06	DOWN
NONMMUT145026.1	2.733938	0.00075	UP	-2.22543	0.005502	DOWN
NONMMUT144862.1	2.825872	0.000763	UP	-3.02175	0.000339	DOWN
NONMMUT098269.1	2.306565	0.000783	UP	-2.86005	2.20E-09	DOWN
ENSMUST00000153523	Inf	0.000829	UP	#NAME?	0.001224	DOWN
NONMMUT148959.1	2.39635	0.000835	UP	-1.82185	0.022286	DOWN
NONMMUT082612.1	2.32774	0.000862	UP	-2.21433	0.00079	DOWN
NONMMUT085423.1	4.481145	0.000912	UP	-2.59391	0.027547	DOWN
NONMMUT014906.2	3.495239	0.000975	UP	-3.165	0.00141	DOWN
NONMMUT085957.1	Inf	0.001003	UP	#NAME?	0.007888	DOWN
NONMMUT012249.2	Inf	0.001048	UP	#NAME?	0.019647	DOWN
NONMMUT016779.2	Inf	0.001071	UP	#NAME?	0.000103	DOWN
NONMMUT111665.1	3.400352	0.001093	UP	-3.00848	0.016469	DOWN
NONMMUT058911.2	Inf	0.001127	UP	#NAME?	0.000117	DOWN
ENSMUST00000143062	Inf	0.001229	UP	#NAME?	0.000741	DOWN
NONMMUT002357.2	1.272545	0.001281	UP	-1.8853	1.10E-05	DOWN
NONMMUT145911.1	2.259063	0.001416	UP	-3.00013	0.000406	DOWN
NONMMUT005462.2	2.188452	0.00144	UP	-3.9247	0.00019	DOWN
ENSMUST00000217740	Inf	0.001482	UP	#NAME?	0.03787	DOWN
NONMMUT105664.1	Inf	0.001512	UP	#NAME?	0.000196	DOWN
NONMMUT084772.1	2.137965	0.001525	UP	-1.98444	0.030975	DOWN
NONMMUT086698.1	Inf	0.001579	UP	#NAME?	0.040762	DOWN
NONMMUT026756.2	Inf	0.001593	UP	#NAME?	3.79E-06	DOWN
ENSMUST00000203139	Inf	0.001634	UP	#NAME?	0.028069	DOWN
NONMMUT106058.1	Inf	0.001682	UP	#NAME?	0.010172	DOWN
ENSMUST00000123107	Inf	0.001698	UP	#NAME?	0.009351	DOWN
NONMMUT038181.2	4.125149	0.001706	UP	-3.51717	0.022306	DOWN
NONMMUT037357.2	1.44906	0.001709	UP	-1.20806	0.009489	DOWN
NONMMUT083810.1	Inf	0.001728	UP	#NAME?	0.016559	DOWN
NONMMUT151976.1	4.378227	0.001742	UP	-3.14194	0.033452	DOWN
NONMMUT105903.1	Inf	0.001743	UP	#NAME?	0.008704	DOWN
NONMMUT029108.2	1.641602	0.001868	UP	-1.54454	0.000248	DOWN
NONMMUT049631.2	2.506348	0.002162	UP	-2.9345	7.23E-05	DOWN
NONMMUT144900.1	Inf	0.002369	UP	#NAME?	0.005118	DOWN
ENSMUST00000214407	1.637673	0.002554	UP	-1.41971	0.011903	DOWN
NONMMUT139410.1	2.338117	0.002568	UP	-2.88884	5.32E-05	DOWN
NONMMUT071137.2	4.175508	0.002584	UP	-4.68653	0.000105	DOWN
NONMMUT036416.2	Inf	0.002669	UP	#NAME?	0.014499	DOWN
NONMMUT032182.2	3.771952	0.00274	UP	-3.30598	0.026817	DOWN
NONMMUT067433.2	2.503929	0.002851	UP	-2.56721	0.036996	DOWN
NONMMUT127860.1	3.826814	0.003062	UP	-4.39494	3.11E-05	DOWN
NONMMUT065814.2	2.477422	0.003249	UP	-2.56245	0.000742	DOWN
ENSMUST00000181107	3.403546	0.003277	UP	-2.84038	0.01362	DOWN
NONMMUT150177.1	Inf	0.003285	UP	#NAME?	0.001409	DOWN
NONMMUT068574.2	2.191468	0.003364	UP	-1.53897	0.002069	DOWN

NONMMUT050471.2	1.145762	0.00341	UP	-1.23538	0.003924	DOWN
NONMMUT064747.2	1.628943	0.003478	UP	-1.65537	0.008543	DOWN
NONMMUT051328.2	2.5138	0.003531	UP	-3.39097	0.003536	DOWN
NONMMUT007491.2	3.722279	0.003671	UP	-3.17986	0.020863	DOWN
NONMMUT052915.2	3.783289	0.003684	UP	-4.27164	0.001626	DOWN
NONMMUT095141.1	3.430743	0.003838	UP	-3.09183	0.004947	DOWN
NONMMUT070299.2	1.954348	0.003848	UP	-2.10696	0.008798	DOWN
NONMMUT009784.2	3.365395	0.00385	UP	-3.07821	0.005722	DOWN
NONMMUT032853.2	3.056726	0.003879	UP	-3.01215	0.003212	DOWN
NONMMUT031472.2	2.906945	0.003927	UP	-3.63478	0.000214	DOWN
NONMMUT125491.1	3.072595	0.004025	UP	-3.35565	0.016557	DOWN
NONMMUT005618.2	Inf	0.0041	UP	#NAME?	0.001145	DOWN
NONMMUT121267.1	3.819805	0.004216	UP	-4.60964	0.003848	DOWN
NONMMUT026984.2	3.18429	0.004282	UP	-2.36474	0.047615	DOWN
ENSMUST00000195382	1.149816	0.004313	UP	-1.57691	0.000651	DOWN
ENSMUST00000176925	Inf	0.004451	UP	#NAME?	0.002758	DOWN
NONMMUT006754.2	1.58532	0.00455	UP	-1.40809	0.018624	DOWN
NONMMUT099013.1	Inf	0.004613	UP	#NAME?	0.010808	DOWN
NONMMUT060728.2	Inf	0.004723	UP	#NAME?	2.23E-05	DOWN
NONMMUT144108.1	Inf	0.005022	UP	#NAME?	0.002791	DOWN
NONMMUT008442.2	3.562042	0.00503	UP	-3.16894	0.013451	DOWN
NONMMUT040618.2	3.00204	0.005043	UP	-3.58832	0.004765	DOWN
NONMMUT063320.2	1.650368	0.005153	UP	-4.28466	1.10E-11	DOWN
NONMMUT120980.1	2.43447	0.005198	UP	-3.13403	7.56E-05	DOWN
NONMMUT082609.1	1.936748	0.005276	UP	-2.2665	0.000334	DOWN
ENSMUST00000223248	1.494091	0.005276	UP	-1.43136	0.000788	DOWN
NONMMUT019266.2	1.329356	0.005321	UP	-1.298	0.002153	DOWN
NONMMUT089908.1	1.654509	0.005459	UP	-2.64871	1.23E-05	DOWN
NONMMUT066790.2	3.263898	0.005509	UP	-4.39316	8.31E-05	DOWN
NONMMUT044168.2	3.160743	0.005578	UP	-2.80709	0.013399	DOWN
NONMMUT136361.1	Inf	0.005695	UP	#NAME?	0.007206	DOWN
NONMMUT041831.2	Inf	0.005721	UP	#NAME?	0.007859	DOWN
NONMMUT059199.2	1.635883	0.005727	UP	-2.03162	3.86E-07	DOWN
NONMMUT037666.2	Inf	0.00578	UP	#NAME?	0.005045	DOWN
NONMMUT054434.2	2.944334	0.00584	UP	-3.51352	0.000753	DOWN
ENSMUST00000227412	Inf	0.005913	UP	#NAME?	0.00017	DOWN
NONMMUT083099.1	2.20389	0.005925	UP	-2.50042	0.000639	DOWN
NONMMUT113826.1	3.712397	0.005962	UP	-3.91795	0.004922	DOWN
NONMMUT035289.2	1.263255	0.005974	UP	-1.17578	0.029488	DOWN
NONMMUT036886.2	3.665508	0.006111	UP	-4.65911	0.001803	DOWN
NONMMUT028236.2	3.529686	0.006153	UP	-5.95697	0.000697	DOWN
NONMMUT024511.2	1.261437	0.006363	UP	-3.11136	3.77E-08	DOWN
NONMMUT093617.1	3.191784	0.006582	UP	-3.25771	0.004506	DOWN
NONMMUT121744.1	2.846654	0.006619	UP	-3.22752	0.003622	DOWN
NONMMUT001512.2	Inf	0.006637	UP	#NAME?	0.024908	DOWN
ENSMUST00000211953	Inf	0.006944	UP	#NAME?	6.59E-07	DOWN
NONMMUT045553.2	1.350757	0.007248	UP	-1.6184	0.001062	DOWN
NONMMUT125902.1	3.599458	0.007319	UP	-2.90379	0.042117	DOWN
NONMMUT049630.2	Inf	0.007446	UP	#NAME?	0.000575	DOWN
NONMMUT143553.1	Inf	0.007464	UP	#NAME?	2.82E-05	DOWN

NONMMUT104688.1	1.536419	0.007572	UP	-1.69657	0.009528	DOWN
NONMMUT045554.2	1.30537	0.007689	UP	-1.6113	0.000448	DOWN
NONMMUT071122.2	2.31767	0.007793	UP	-3.66171	1.22E-06	DOWN
NONMMUT070229.2	1.520451	0.008074	UP	-1.58982	0.040095	DOWN
NONMMUT020952.2	3.368	0.00847	UP	-5.00384	2.23E-05	DOWN
ENSMUST00000132210	2.454641	0.008489	UP	-2.37901	0.016161	DOWN
NONMMUT082607.1	1.850278	0.008573	UP	-2.10698	0.002025	DOWN
NONMMUT123243.1	2.326838	0.008645	UP	-2.73882	0.001149	DOWN
NONMMUT021320.2	2.41093	0.008859	UP	-2.7187	6.39E-06	DOWN
NONMMUT114830.1	Inf	0.009072	UP	#NAME?	0.03493	DOWN
NONMMUT000951.2	1.233003	0.009156	UP	-1.01439	0.026041	DOWN
NONMMUT104683.1	2.750878	0.00925	UP	-2.77249	0.002813	DOWN
NONMMUT131139.1	2.742333	0.009284	UP	-2.29765	0.024185	DOWN
NONMMUT104401.1	3.050033	0.009374	UP	-4.82636	1.48E-09	DOWN
NONMMUT020942.2	3.175826	0.009767	UP	-3.42452	0.01013	DOWN
ENSMUST00000207720	2.800268	0.010142	UP	-3.47921	0.000786	DOWN
NONMMUT051843.2	2.625953	0.010303	UP	-2.61691	0.00859	DOWN
NONMMUT093085.1	Inf	0.010372	UP	#NAME?	0.007888	DOWN
NONMMUT058309.2	2.41363	0.01042	UP	-2.23638	0.012358	DOWN
ENSMUST00000210575	1.914039	0.010994	UP	-2.19845	0.01552	DOWN
NONMMUT142459.1	1.397107	0.011084	UP	-1.99963	0.001101	DOWN
NONMMUT023687.2	Inf	0.011344	UP	#NAME?	7.20E-05	DOWN
ENSMUST00000219324	Inf	0.011512	UP	#NAME?	2.48E-05	DOWN
NONMMUT065570.2	1.057041	0.011542	UP	-1.13287	0.012178	DOWN
NONMMUT070129.2	2.376839	0.011613	UP	-2.39772	0.012609	DOWN
NONMMUT006305.2	2.078939	0.011826	UP	-2.16109	0.006773	DOWN
ENSMUST00000144926	Inf	0.012068	UP	#NAME?	0.037222	DOWN
ENSMUST00000142458	1.48483	0.012097	UP	-1.70291	0.013001	DOWN
ENSMUST00000196744	1.952589	0.012109	UP	-2.05917	0.010429	DOWN
NONMMUT145897.1	2.94818	0.012433	UP	-3.58455	0.001757	DOWN
NONMMUT068680.2	1.468927	0.012485	UP	-1.80223	0.001112	DOWN
NONMMUT016573.2	1.21769	0.012689	UP	-1.41692	0.013909	DOWN
NONMMUT024048.2	1.584913	0.012771	UP	-1.55727	0.035241	DOWN
ENSMUST00000228636	1.44874	0.012786	UP	-1.51805	0.027671	DOWN
NONMMUT037309.2	2.380565	0.012854	UP	-2.95902	0.000698	DOWN
NONMMUT145613.1	Inf	0.012929	UP	#NAME?	0.001699	DOWN
NONMMUT097793.1	2.224933	0.012938	UP	-1.98644	0.049892	DOWN
NONMMUT016330.2	1.6396	0.01294	UP	-1.66363	0.031616	DOWN
NONMMUT108397.1	2.297848	0.013143	UP	-2.42159	0.003104	DOWN
NONMMUT021124.2	Inf	0.013584	UP	#NAME?	0.003752	DOWN
ENSMUST00000223261	Inf	0.013586	UP	#NAME?	0.012475	DOWN
NONMMUT121746.1	1.847461	0.013594	UP	-2.92069	0.001475	DOWN
ENSMUST00000201032	2.539905	0.013598	UP	-2.62444	0.013587	DOWN
ENSMUST00000227609	1.329439	0.013936	UP	-1.34269	0.027795	DOWN
NONMMUT027419.2	1.356893	0.013989	UP	-1.62786	0.006307	DOWN
ENSMUST00000194586	1.166962	0.013992	UP	-1.45804	0.002241	DOWN
NONMMUT132335.1	2.182182	0.014126	UP	-2.89194	0.000587	DOWN
ENSMUST00000222965	1.221574	0.014213	UP	-1.81991	3.03E-05	DOWN
NONMMUT067225.2	Inf	0.014409	UP	#NAME?	0.007457	DOWN
NONMMUT112935.1	2.598558	0.014675	UP	-3.12074	0.000742	DOWN

NONMMUT026054.2	1.359795	0.014903	UP	-1.62739	0.00021	DOWN
ENSMUST00000148972	2.186908	0.015264	UP	-3.16589	0.000772	DOWN
NONMMUT079507.1	2.108623	0.015308	UP	-3.10012	0.000344	DOWN
MSTRG.53209.18	1.120936	0.015337	UP	-1.20654	0.018956	DOWN
NONMMUT062890.2	1.157257	0.015395	UP	-3.14328	2.35E-08	DOWN
NONMMUT111094.1	Inf	0.015535	UP	#NAME?	0.000318	DOWN
NONMMUT041160.2	Inf	0.015736	UP	#NAME?	0.013811	DOWN
NONMMUT112066.1	Inf	0.015736	UP	#NAME?	0.007888	DOWN
NONMMUT144224.1	Inf	0.015736	UP	#NAME?	0.004035	DOWN
NONMMUT114119.1	2.892291	0.016488	UP	-3.06719	0.02221	DOWN
NONMMUT101832.1	2.197522	0.016743	UP	-2.77239	0.001038	DOWN
NONMMUT041595.2	3.269557	0.016857	UP	-3.35469	0.017942	DOWN
ENSMUST00000217680	3.052595	0.016861	UP	-2.85681	0.035896	DOWN
NONMMUT104385.1	Inf	0.016904	UP	#NAME?	0.020406	DOWN
NONMMUT090042.1	Inf	0.017147	UP	#NAME?	0.017198	DOWN
NONMMUT028948.2	1.414559	0.017267	UP	-1.85332	0.010549	DOWN
NONMMUT142849.1	1.781112	0.01733	UP	-1.97334	0.010924	DOWN
NONMMUT048462.2	Inf	0.017517	UP	#NAME?	0.000934	DOWN
NONMMUT100922.1	1.271057	0.018234	UP	-1.51266	0.007698	DOWN
NONMMUT148890.1	3.205156	0.018613	UP	-3.37594	0.037309	DOWN
NONMMUT009222.2	1.880118	0.018735	UP	-2.89157	5.64E-05	DOWN
ENSMUST00000190917	1.491142	0.019678	UP	-1.95412	0.016418	DOWN
NONMMUT052905.2	Inf	0.01969	UP	#NAME?	0.047348	DOWN
NONMMUT093070.1	Inf	0.019775	UP	#NAME?	0.007834	DOWN
NONMMUT102759.1	Inf	0.01987	UP	#NAME?	0.033254	DOWN
NONMMUT103293.1	3.328263	0.019947	UP	-3.29807	0.028639	DOWN
NONMMUT148899.1	1.854428	0.019961	UP	-1.82669	0.000994	DOWN
NONMMUT120979.1	2.207608	0.019966	UP	-3.00306	0.000321	DOWN
ENSMUST00000211335	1.795249	0.020051	UP	-1.6942	0.044377	DOWN
NONMMUT020956.2	3.309846	0.020601	UP	-4.81189	3.88E-05	DOWN
NONMMUT096189.1	1.262365	0.020664	UP	-1.46622	0.003825	DOWN
NONMMUT020954.2	2.639729	0.02073	UP	-3.88388	7.55E-05	DOWN
NONMMUT019488.2	1.244737	0.021109	UP	-2.2773	2.55E-05	DOWN
NONMMUT089907.1	1.397039	0.021163	UP	-2.29617	0.000239	DOWN
NONMMUT001616.2	2.299814	0.021216	UP	-2.00344	0.013281	DOWN
ENSMUST00000181558	2.115522	0.021269	UP	-2.50014	0.002611	DOWN
NONMMUT036933.2	2.150073	0.0213	UP	-1.98392	0.043421	DOWN
NONMMUT018610.2	1.275341	0.021775	UP	-1.56092	0.006534	DOWN
NONMMUT092674.1	1.649994	0.021812	UP	-2.01352	0.001921	DOWN
ENSMUST00000137752	Inf	0.022053	UP	#NAME?	0.015197	DOWN
NONMMUT068568.2	Inf	0.022132	UP	#NAME?	1.61E-05	DOWN
NONMMUT086230.1	1.444709	0.022513	UP	-1.43151	0.036259	DOWN
NONMMUT107279.1	1.306418	0.023388	UP	-1.56899	0.00897	DOWN
NONMMUT000680.2	2.143756	0.023535	UP	-2.396	0.006349	DOWN
NONMMUT129191.1	Inf	0.023649	UP	#NAME?	0.047208	DOWN
NONMMUT055764.2	2.320391	0.023805	UP	-2.68781	0.010636	DOWN
ENSMUST00000160126	3.089119	0.023942	UP	-4.84242	9.73E-08	DOWN
NONMMUT019257.2	1.138787	0.024022	UP	-1.28237	0.006042	DOWN
NONMMUT004318.2	1.118358	0.024177	UP	-1.54841	0.016731	DOWN
NONMMUT081472.1	3.669301	0.024628	UP	-3.76661	0.02199	DOWN

ENSMUST00000132264	2.266873	0.024752	UP	-2.52278	0.012791	DOWN
NONMMUT027946.2	2.244892	0.024848	UP	-2.55158	0.002052	DOWN
NONMMUT104682.1	2.09585	0.024933	UP	-2.27375	0.01592	DOWN
NONMMUT083995.1	1.824743	0.025293	UP	-3.33095	0.006818	DOWN
NONMMUT091482.1	2.692997	0.0255	UP	-2.92279	0.044019	DOWN
NONMMUT046059.2	2.479987	0.026006	UP	-2.42446	0.033271	DOWN
NONMMUT093071.1	Inf	0.026227	UP	#NAME?	0.049857	DOWN
NONMMUT131176.1	2.82718	0.026265	UP	-4.10634	6.06E-05	DOWN
NONMMUT132620.1	2.437679	0.02642	UP	-2.8615	0.021669	DOWN
NONMMUT005779.2	Inf	0.026479	UP	#NAME?	0.006717	DOWN
NONMMUT117756.1	1.32476	0.0265	UP	-1.9721	0.001235	DOWN
ENSMUST00000194151	1.028205	0.026848	UP	-1.18824	0.022333	DOWN
NONMMUT053851.2	Inf	0.027378	UP	#NAME?	0.001885	DOWN
NONMMUT142245.1	1.799837	0.027559	UP	-1.76264	0.008112	DOWN
NONMMUT104690.1	1.41436	0.027741	UP	-1.80513	0.00522	DOWN
NONMMUT069137.2	1.051031	0.027877	UP	-1.1903	0.031487	DOWN
NONMMUT067083.2	2.30268	0.02796	UP	-5.42088	3.93E-05	DOWN
NONMMUT042458.2	Inf	0.027988	UP	#NAME?	0.015702	DOWN
NONMMUT056702.2	1.839646	0.028058	UP	-2.61329	0.001844	DOWN
NONMMUT124526.1	Inf	0.02817	UP	#NAME?	0.024801	DOWN
NONMMUT049261.2	1.38495	0.028981	UP	-1.66311	0.027422	DOWN
ENSMUST00000151112	Inf	0.029616	UP	#NAME?	0.000168	DOWN
NONMMUT033808.2	Inf	0.029617	UP	#NAME?	0.000827	DOWN
ENSMUST00000152627	Inf	0.029709	UP	#NAME?	0.0466	DOWN
NONMMUT071374.2	2.210356	0.030232	UP	-2.63896	0.0145	DOWN
NONMMUT081313.1	Inf	0.030705	UP	#NAME?	0.004117	DOWN
NONMMUT044870.2	1.007668	0.030911	UP	-1.59105	0.000639	DOWN
NONMMUT093720.1	Inf	0.031024	UP	#NAME?	0.003679	DOWN
NONMMUT132192.1	3.246805	0.031323	UP	-3.52515	0.036828	DOWN
NONMMUT012041.2	1.737659	0.031347	UP	-2.00465	0.02383	DOWN
NONMMUT040063.2	1.049302	0.031985	UP	-1.60714	0.002436	DOWN
ENSMUST00000227739	Inf	0.032041	UP	#NAME?	0.022892	DOWN
NONMMUT124607.1	Inf	0.032045	UP	#NAME?	0.000265	DOWN
NONMMUT043997.2	1.985835	0.032962	UP	-3.46379	2.67E-10	DOWN
NONMMUT063321.2	1.392027	0.033136	UP	-3.82784	8.54E-09	DOWN
ENSMUST00000228259	2.075865	0.033165	UP	-2.90785	0.009192	DOWN
ENSMUST00000196108	1.259275	0.033281	UP	-1.96656	0.001518	DOWN
ENSMUST00000200033	1.075319	0.033532	UP	-1.53137	0.004913	DOWN
NONMMUT067703.2	1.986325	0.034259	UP	-4.05002	0.000195	DOWN
NONMMUT045906.2	2.124682	0.035026	UP	-2.55675	0.005425	DOWN
NONMMUT006595.2	2.722761	0.035391	UP	-4.13016	0.000508	DOWN
NONMMUT079501.1	1.471694	0.035519	UP	-3.14191	1.26E-05	DOWN
NONMMUT113863.1	1.179964	0.035829	UP	-1.60869	0.006883	DOWN
NONMMUT007296.2	2.014255	0.035886	UP	-1.82538	0.04872	DOWN
NONMMUT051867.2	1.042047	0.035917	UP	-1.58028	0.003522	DOWN
NONMMUT027151.2	2.173588	0.036251	UP	-2.50727	0.038424	DOWN
NONMMUT048676.2	1.8182	0.036268	UP	-2.23584	0.034269	DOWN
ENSMUST00000228514	Inf	0.036283	UP	#NAME?	7.05E-05	DOWN
NONMMUT049629.2	2.256828	0.037615	UP	-2.86248	0.007747	DOWN
NONMMUT028049.2	4.423993	0.038034	UP	-5.47519	0.000103	DOWN

NONMMUT074302.2	1.32386	0.038176	UP		-2.3364	0.000519	DOWN
ENSMUST00000181218	2.58031	0.038236	UP		-2.79082	0.024143	DOWN
NONMMUT098272.1	2.568467	0.038263	UP		-2.645	0.031711	DOWN
NONMMUT003505.2	2.794479	0.038341	UP		-4.24781	7.72E-05	DOWN
NONMMUT018368.2	1.033446	0.03847	UP		-1.58815	0.000253	DOWN
NONMMUT018269.2	Inf	0.038625	UP		#NAME?	0.003706	DOWN
NONMMUT020640.2	2.052915	0.038643	UP		-2.17512	0.040659	DOWN
NONMMUT032158.2	1.810016	0.038683	UP		-2.49792	0.002164	DOWN
NONMMUT109465.1	3.209766	0.039348	UP		-3.5898	0.026269	DOWN
NONMMUT122583.1	Inf	0.039421	UP		#NAME?	0.000266	DOWN
NONMMUT055714.2	1.017915	0.039856	UP		-1.07938	0.031784	DOWN
NONMMUT033627.2	2.051816	0.040011	UP		-3.39293	0.000627	DOWN
ENSMUST00000140716	1.247507	0.040295	UP		-1.46306	0.031221	DOWN
ENSMUST00000192154	1.011855	0.040541	UP		-1.05223	0.033602	DOWN
ENSMUST00000206384	1.073792	0.04063	UP		-1.95959	0.000162	DOWN
NONMMUT035346.2	1.663925	0.040809	UP		-3.39956	0.001576	DOWN
NONMMUT104689.1	1.404215	0.040926	UP		-1.74739	0.015553	DOWN
NONMMUT091098.1	Inf	0.040996	UP		#NAME?	0.027357	DOWN
NONMMUT151902.1	1.261113	0.041268	UP		-1.61687	0.03155	DOWN
NONMMUT091662.1	Inf	0.041357	UP		#NAME?	0.014278	DOWN
NONMMUT043068.2	Inf	0.04138	UP		#NAME?	0.001409	DOWN
NONMMUT055718.2	1.150125	0.041578	UP		-1.24528	0.037935	DOWN
ENSMUST00000126622	Inf	0.041998	UP		#NAME?	0.004343	DOWN
NONMMUT145530.1	Inf	0.042211	UP		#NAME?	0.043647	DOWN
NONMMUT033453.2	3.547104	0.042324	UP		-3.99847	0.000422	DOWN
NONMMUT106467.1	1.190779	0.042331	UP		-1.53898	0.024878	DOWN
NONMMUT009255.2	1.820977	0.042637	UP		-2.22689	0.010467	DOWN
NONMMUT104696.1	1.323887	0.043169	UP		-1.67233	0.010694	DOWN
ENSMUST00000219954	Inf	0.043171	UP		#NAME?	0.010472	DOWN
NONMMUT082665.1	2.580492	0.043215	UP		-2.99797	0.003514	DOWN
NONMMUT147625.1	Inf	0.043246	UP		#NAME?	0.004034	DOWN
NONMMUT130037.1	1.705262	0.043256	UP		-1.76109	0.035188	DOWN
ENSMUST00000192025	1.035109	0.043304	UP		-1.32791	0.002174	DOWN
NONMMUT043074.2	Inf	0.043387	UP		#NAME?	0.046031	DOWN
NONMMUT114839.1	Inf	0.04374	UP		#NAME?	0.000579	DOWN
NONMMUT058329.2	3.11998	0.043859	UP		-3.34589	0.022436	DOWN
NONMMUT121745.1	1.661402	0.044555	UP		-2.90706	0.001595	DOWN
NONMMUT025800.2	1.152777	0.044875	UP		-1.56139	0.004002	DOWN
NONMMUT040060.2	1.015018	0.044876	UP		-1.19797	0.047115	DOWN
NONMMUT137557.1	1.042899	0.045192	UP		-1.33318	0.009757	DOWN
ENSMUST00000224036	1.060076	0.045599	UP		-1.22111	0.036314	DOWN
NONMMUT000952.2	1.168553	0.04562	UP		-1.10758	0.016258	DOWN
NONMMUT066463.2	1.225205	0.045685	UP		-1.52706	0.003906	DOWN
ENSMUST00000157948	1.514458	0.04587	UP		-1.9593	0.036083	DOWN
NONMMUT080947.1	3.545138	0.047851	UP		-3.51511	0.025352	DOWN
NONMMUT092900.1	1.443286	0.048115	UP		-2.05571	0.008372	DOWN
NONMMUT051468.2	2.554065	0.048154	UP		-3.56141	0.000116	DOWN
NONMMUT002689.2	1.256229	0.048472	UP		-2.01203	0.004747	DOWN
NONMMUT128858.1	1.143397	0.048488	UP		-1.94057	0.002016	DOWN
NONMMUT085167.1	2.536311	0.048499	UP		-2.68894	0.046929	DOWN

ENSMUST00000120178	1.345769	0.048655	UP	-1.58888	0.03242	DOWN
NONMMUT077089.1	1.730232	0.0487	UP	-2.00461	0.020569	DOWN
NONMMUT071806.2	1.725382	0.049027	UP	-2.44897	0.000908	DOWN
NONMMUT027438.2	2.776355	0.049101	UP	-3.44562	0.002964	DOWN
NONMMUT104686.1	1.346655	0.049355	UP	-1.47721	0.028232	DOWN
NONMMUT082603.1	1.477762	0.04937	UP	-1.85876	0.012445	DOWN
NONMMUT040147.2	1.211655	0.049533	UP	-1.56577	0.024596	DOWN
NONMMUT082605.1	2.641581	0.049595	UP	-3.12696	0.015624	DOWN
NONMMUT021269.2	1.928656	0.049657	UP	-2.79194	0.005248	DOWN
NONMMUT110591.1	3.076259	0.049663	UP	-3.11296	0.022791	DOWN
NONMMUT020278.2	1.388825	0.049701	UP	-1.86525	0.003759	DOWN
NONMMUT055598.2	1.019088	0.049733	UP	-1.52029	0.005043	DOWN
NONMMUT080439.1	3.042139	0.049876	UP	-4.01655	0.002409	DOWN
NONMMUT018494.2	-6.63171	1.25E-30	DOWN	6.72612	2.72E-30	UP
NONMMUT071342.2	-3.80688	1.60E-13	DOWN	1.630474	0.012079	UP
NONMMUT153460.1	-4.43375	1.79E-11	DOWN	3.98791	1.14E-10	UP
NONMMUT146601.1	#NAME?	7.51E-10	DOWN	Inf	7.93E-10	UP
NONMMUT006953.2	-2.36716	2.40E-09	DOWN	1.275955	0.000623	UP
NONMMUT144113.1	#NAME?	3.22E-09	DOWN	Inf	3.20E-09	UP
NONMMUT070926.2	-2.66561	8.38E-08	DOWN	1.344882	0.026106	UP
NONMMUT068026.2	#NAME?	1.20E-07	DOWN	3.220757	0.001089	UP
NONMMUT019242.2	-1.86027	2.37E-07	DOWN	2.994019	1.34E-16	UP
NONMMUT139818.1	-2.93598	3.09E-07	DOWN	4.275103	5.42E-10	UP
ENSMUST00000145549	-1.97828	7.99E-07	DOWN	1.981088	3.80E-07	UP
NONMMUT001029.2	-6.20954	1.09E-06	DOWN	5.951074	1.73E-06	UP
NONMMUT047957.2	-2.17202	1.80E-06	DOWN	3.62283	5.31E-12	UP
NONMMUT056994.2	-3.97586	2.22E-06	DOWN	4.330643	1.11E-07	UP
NONMMUT041793.2	-4.15666	2.47E-06	DOWN	3.348028	0.000102	UP
NONMMUT015752.2	#NAME?	5.27E-06	DOWN	Inf	4.75E-06	UP
NONMMUT006717.2	-2.02194	8.66E-06	DOWN	2.041246	1.41E-06	UP
NONMMUT044528.2	-4.67614	1.12E-05	DOWN	4.314995	2.45E-05	UP
NONMMUT028282.2	#NAME?	1.19E-05	DOWN	3.738517	0.037323	UP
MSTRG.26789.5	-2.35799	1.25E-05	DOWN	3.346146	4.90E-17	UP
NONMMUT005295.2	-1.41758	2.03E-05	DOWN	1.449639	1.11E-06	UP
NONMMUT044897.2	-1.43116	2.19E-05	DOWN	1.249501	2.60E-05	UP
NONMMUT147944.1	-7.65705	2.59E-05	DOWN	7.361654	3.08E-05	UP
NONMMUT006490.2	-4.25073	3.25E-05	DOWN	4.040499	4.37E-05	UP
NONMMUT141647.1	-3.7519	3.30E-05	DOWN	3.045933	2.40E-05	UP
NONMMUT054892.2	-1.99848	3.44E-05	DOWN	2.304363	8.76E-08	UP
NONMMUT051479.2	-3.05151	3.84E-05	DOWN	2.213055	0.00098	UP
NONMMUT034722.2	-5.99116	5.98E-05	DOWN	5.925451	5.74E-05	UP
MSTRG.7055.12	-7.5669	6.81E-05	DOWN	Inf	1.75E-06	UP
NONMMUT042491.2	-6.0331	6.81E-05	DOWN	5.439455	0.000152	UP
NONMMUT028972.2	-5.33988	7.69E-05	DOWN	5.109498	9.40E-05	UP
NONMMUT003238.2	-5.56027	8.43E-05	DOWN	2.187198	0.045286	UP
NONMMUT144881.1	#NAME?	8.88E-05	DOWN	3.119747	0.01192	UP
NONMMUT091545.1	#NAME?	0.000101	DOWN	Inf	0.000101	UP
NONMMUT056862.2	-6.47057	0.000104	DOWN	6.372533	0.000101	UP
NONMMUT061044.2	-5.02265	0.000116	DOWN	3.580173	0.000913	UP
NONMMUT011659.2	-5.39538	0.000122	DOWN	5.319133	0.000118	UP

NONMMUT004274.2	-1.86821	0.000128	DOWN	2.077963	1.51E-05	UP
NONMMUT033251.2	-2.82455	0.000132	DOWN	1.433706	0.004868	UP
NONMMUT051032.2	-3.63123	0.000134	DOWN	2.978402	0.00045	UP
NONMMUT056993.2	-3.80737	0.000155	DOWN	3.254803	0.000543	UP
NONMMUT139779.1	#NAME?	0.000163	DOWN	Inf	0.000161	UP
ENSMUST00000161091	#NAME?	0.00019	DOWN	Inf	0.00019	UP
NONMMUT000506.2	-2.00412	0.0002	DOWN	2.02832	7.57E-05	UP
NONMMUT006943.2	-2.69896	0.000202	DOWN	3.491701	7.64E-06	UP
NONMMUT074194.2	-1.40129	0.000222	DOWN	1.216793	0.001626	UP
NONMMUT142323.1	#NAME?	0.000229	DOWN	2.671541	0.033854	UP
NONMMUT001113.2	-5.03622	0.000237	DOWN	5.062797	0.000211	UP
NONMMUT127646.1	-1.49991	0.000247	DOWN	1.664479	3.18E-05	UP
NONMMUT153461.1	#NAME?	0.000256	DOWN	5.284574	0.001038	UP
NONMMUT017412.2	-4.16121	0.000271	DOWN	4.083163	0.000253	UP
NONMMUT109311.1	#NAME?	0.000346	DOWN	Inf	0.000329	UP
NONMMUT140130.1	#NAME?	0.000401	DOWN	2.60771	0.04963	UP
NONMMUT021492.2	-1.73628	0.000403	DOWN	1.33823	0.004408	UP
NONMMUT075890.1	#NAME?	0.000414	DOWN	1.920766	0.03266	UP
NONMMUT023599.2	-3.77067	0.000417	DOWN	3.066497	0.001738	UP
NONMMUT140771.1	#NAME?	0.000422	DOWN	Inf	0.000411	UP
NONMMUT011499.2	-3.08549	0.00043	DOWN	1.938894	0.025245	UP
NONMMUT045059.2	-2.31426	0.000455	DOWN	1.818992	0.002051	UP
NONMMUT064551.2	-3.77916	0.000508	DOWN	3.682119	0.000469	UP
NONMMUT051900.2	-4.20605	0.000511	DOWN	4.223767	0.000392	UP
NONMMUT072529.2	-3.65903	0.000519	DOWN	3.711007	0.000352	UP
NONMMUT026006.2	-1.27756	0.000545	DOWN	1.71167	1.23E-06	UP
NONMMUT021110.2	-3.98154	0.000555	DOWN	4.062078	0.000367	UP
NONMMUT009671.2	-1.85259	0.000642	DOWN	1.658183	0.000962	UP
NONMMUT000762.2	-3.48238	0.000643	DOWN	3.294464	0.000777	UP
NONMMUT025824.2	-4.64877	0.000698	DOWN	4.502524	0.000721	UP
NONMMUT037802.2	-3.55091	0.000758	DOWN	3.689116	0.000427	UP
NONMMUT031873.2	-1.32875	0.000809	DOWN	1.000675	0.002708	UP
NONMMUT067945.2	-2.42029	0.000831	DOWN	2.004896	0.003776	UP
NONMMUT115247.1	-2.24081	0.00085	DOWN	2.974202	3.48E-06	UP
NONMMUT068214.2	-3.29625	0.000857	DOWN	3.426508	0.000473	UP
NONMMUT041462.2	-3.14546	0.000891	DOWN	5.040669	3.03E-05	UP
NONMMUT034730.2	-3.73031	0.000895	DOWN	3.759188	0.000544	UP
NONMMUT005179.2	-3.16609	0.001035	DOWN	2.912987	0.001585	UP
ENSMUST00000205632	-2.06403	0.001124	DOWN	1.819923	0.001629	UP
NONMMUT018487.2	-3.45077	0.001125	DOWN	3.400361	0.0011	UP
NONMMUT146291.1	#NAME?	0.001198	DOWN	Inf	0.001118	UP
NONMMUT068190.2	-2.18228	0.001209	DOWN	1.211794	0.031069	UP
NONMMUT018276.2	#NAME?	0.001213	DOWN	Inf	0.001161	UP
NONMMUT152443.1	-3.13908	0.001265	DOWN	2.415444	0.00262	UP
NONMMUT006942.2	-1.07123	0.001282	DOWN	1.368786	5.38E-06	UP
NONMMUT088282.1	-2.52215	0.001382	DOWN	3.280079	0.000103	UP
NONMMUT063916.2	-4.04223	0.00158	DOWN	3.70671	0.002252	UP
NONMMUT074195.2	-1.21041	0.001734	DOWN	1.252913	0.001806	UP
NONMMUT114120.1	-1.70389	0.001773	DOWN	1.469136	0.005179	UP
NONMMUT018145.2	-1.25142	0.00185	DOWN	1.080689	0.001379	UP

NONMMUT038154.2	-1.14292	0.001863	DOWN	1.545779	1.35E-05	UP
NONMMUT030429.2	#NAME?	0.002048	DOWN	Inf	0.001754	UP
NONMMUT072370.2	#NAME?	0.0021	DOWN	Inf	0.002011	UP
NONMMUT004229.2	-4.24796	0.002149	DOWN	2.973688	0.009163	UP
NONMMUT150791.1	-4.8424	0.002321	DOWN	Inf	0.000159	UP
NONMMUT019241.2	-1.24145	0.002356	DOWN	2.736213	4.78E-12	UP
ENSMUST00000098867	-2.32897	0.002486	DOWN	1.8774	0.005676	UP
NONMMUT037200.2	-2.79841	0.00249	DOWN	2.875865	0.001592	UP
NONMMUT085019.1	-3.12101	0.002532	DOWN	2.101283	0.012061	UP
NONMMUT022456.2	#NAME?	0.002779	DOWN	Inf	0.002725	UP
NONMMUT009926.2	-1.14466	0.002811	DOWN	1.089468	0.001135	UP
NONMMUT075885.1	-2.60242	0.002871	DOWN	3.365076	0.000448	UP
ENSMUST00000130744	#NAME?	0.002964	DOWN	Inf	0.003021	UP
ENSMUST00000128094	-1.68169	0.003025	DOWN	1.709211	0.005461	UP
NONMMUT064818.2	-5.57111	0.003128	DOWN	Inf	0.000909	UP
NONMMUT104801.1	#NAME?	0.003225	DOWN	Inf	0.003141	UP
ENSMUST00000222396	-4.72369	0.00338	DOWN	3.739426	0.007829	UP
NONMMUT058032.2	-1.13702	0.003661	DOWN	1.606575	0.000116	UP
NONMMUT105841.1	#NAME?	0.003665	DOWN	Inf	0.003651	UP
MSTRG.15728.1	-1.39301	0.003684	DOWN	2.761881	2.46E-08	UP
NONMMUT013066.2	-1.99167	0.003724	DOWN	1.111856	0.015469	UP
ENSMUST00000135366	-3.89432	0.00382	DOWN	2.869338	0.014005	UP
NONMMUT141897.1	#NAME?	0.003922	DOWN	2.419484	0.044289	UP
NONMMUT051975.2	-2.71744	0.003932	DOWN	2.362953	0.00702	UP
NONMMUT034442.2	-1.55463	0.003941	DOWN	1.228505	0.009137	UP
ENSMUST00000202307	#NAME?	0.003947	DOWN	3.18627	0.033887	UP
ENSMUST00000181291	#NAME?	0.003948	DOWN	3.434683	0.031123	UP
NONMMUT098746.1	#NAME?	0.003954	DOWN	Inf	0.003955	UP
NONMMUT047958.2	-1.57592	0.003955	DOWN	2.377177	5.34E-05	UP
NONMMUT010630.2	-3.58138	0.003982	DOWN	3.483338	0.003813	UP
NONMMUT063720.2	#NAME?	0.004199	DOWN	Inf	0.004296	UP
NONMMUT017414.2	-3.5391	0.004437	DOWN	3.408242	0.004885	UP
ENSMUST00000212899	-1.17873	0.0045	DOWN	1.017475	0.009512	UP
NONMMUT115240.1	-1.7301	0.005036	DOWN	3.025472	8.23E-06	UP
NONMMUT145102.1	-4.95929	0.005152	DOWN	5.352016	0.004538	UP
NONMMUT004166.2	-1.13714	0.005154	DOWN	1.305941	1.84E-05	UP
NONMMUT115224.1	-2.2353	0.005177	DOWN	3.549763	0.000298	UP
NONMMUT034171.2	-2.64504	0.005187	DOWN	2.713172	0.003505	UP
NONMMUT141643.1	#NAME?	0.005291	DOWN	Inf	0.005363	UP
NONMMUT115232.1	-2.48758	0.005429	DOWN	4.169767	8.52E-05	UP
NONMMUT032686.2	-2.35022	0.005582	DOWN	3.025167	0.00084	UP
NONMMUT010546.2	-2.09747	0.005796	DOWN	1.170747	0.049494	UP
NONMMUT032822.2	#NAME?	0.005804	DOWN	Inf	0.005432	UP
ENSMUST00000212427	-2.47797	0.005898	DOWN	3.034692	0.001004	UP
NONMMUT021423.2	-2.70485	0.006005	DOWN	2.581883	0.006565	UP
NONMMUT006364.2	-2.86982	0.006089	DOWN	2.355512	0.012342	UP
NONMMUT144296.1	-1.18101	0.006191	DOWN	2.202638	1.14E-07	UP
NONMMUT068192.2	-2.24585	0.006398	DOWN	1.531044	0.028492	UP
NONMMUT026957.2	-2.26488	0.006793	DOWN	2.381647	0.003016	UP
NONMMUT144896.1	#NAME?	0.007183	DOWN	Inf	0.00712	UP

NONMMUT140755.1	#NAME?	0.00721	DOWN	3.016593	0.034201	UP
ENSMUST00000223171	-2.46538	0.007351	DOWN	Inf	3.63E-05	UP
NONMMUT115259.1	-2.49484	0.007496	DOWN	2.211146	0.027957	UP
NONMMUT003170.2	-2.48124	0.007499	DOWN	2.506239	0.001757	UP
NONMMUT115254.1	-3.23617	0.007762	DOWN	2.96316	0.009588	UP
NONMMUT084736.1	-3.52452	0.007819	DOWN	2.509293	0.024593	UP
NONMMUT004062.2	-2.26037	0.007863	DOWN	2.313781	0.004392	UP
NONMMUT091547.1	-1.90232	0.007881	DOWN	2.575591	0.000423	UP
NONMMUT104643.1	-2.72659	0.008072	DOWN	2.70166	0.007992	UP
NONMMUT061039.2	#NAME?	0.008154	DOWN	Inf	0.007949	UP
ENSMUST00000181114	#NAME?	0.008637	DOWN	Inf	0.008925	UP
NONMMUT115218.1	-1.6025	0.008691	DOWN	3.606837	2.07E-08	UP
NONMMUT115198.1	-1.23036	0.008831	DOWN	4.580441	8.73E-10	UP
NONMMUT078523.1	#NAME?	0.009029	DOWN	2.590141	0.045194	UP
NONMMUT153718.1	#NAME?	0.009039	DOWN	Inf	0.008343	UP
NONMMUT006395.2	-1.53218	0.009193	DOWN	1.212361	0.016804	UP
ENSMUST00000137567	-1.03429	0.009374	DOWN	1.509992	0.000381	UP
NONMMUT024866.2	#NAME?	0.009454	DOWN	Inf	0.009698	UP
MSTRG.7168.3	#NAME?	0.009907	DOWN	Inf	0.009666	UP
NONMMUT041242.2	#NAME?	0.010112	DOWN	Inf	0.009683	UP
NONMMUT144674.1	-3.76685	0.010121	DOWN	Inf	0.002221	UP
NONMMUT071995.2	-1.60754	0.010442	DOWN	1.0675	0.041612	UP
NONMMUT035148.2	-2.07747	0.010453	DOWN	1.784039	0.01356	UP
NONMMUT147206.1	#NAME?	0.010767	DOWN	Inf	0.010846	UP
NONMMUT079620.1	#NAME?	0.01091	DOWN	2.73129	0.047196	UP
NONMMUT115251.1	-1.66413	0.011248	DOWN	4.037978	9.61E-06	UP
ENSMUST00000214762	-1.43569	0.011666	DOWN	2.62091	5.97E-05	UP
NONMMUT031976.2	-2.96119	0.011808	DOWN	Inf	3.85E-05	UP
NONMMUT046460.2	-2.93767	0.011913	DOWN	3.921203	0.003159	UP
ENSMUST00000221520	-1.36377	0.012063	DOWN	2.399688	7.33E-05	UP
NONMMUT045383.2	-2.07803	0.012187	DOWN	1.799528	0.016028	UP
NONMMUT045262.2	-2.63042	0.012372	DOWN	2.521648	0.012907	UP
ENSMUST00000156240	-2.12032	0.012849	DOWN	Inf	7.03E-05	UP
NONMMUT057635.2	#NAME?	0.012904	DOWN	Inf	0.012962	UP
NONMMUT109604.1	-1.41453	0.013123	DOWN	2.107397	0.001584	UP
NONMMUT147892.1	#NAME?	0.013291	DOWN	Inf	0.013112	UP
NONMMUT100573.1	-3.37522	0.013306	DOWN	2.350237	0.045762	UP
NONMMUT107951.1	-1.01877	0.013391	DOWN	1.369976	0.000296	UP
NONMMUT037646.2	-2.07103	0.013464	DOWN	1.987113	0.013712	UP
NONMMUT038697.2	-1.49045	0.014137	DOWN	1.121044	0.023481	UP
NONMMUT115257.1	-1.67765	0.014243	DOWN	2.67089	0.000625	UP
NONMMUT022600.2	-1.25004	0.014354	DOWN	2.072973	1.04E-06	UP
NONMMUT115228.1	-1.43165	0.014447	DOWN	1.902608	0.001059	UP
NONMMUT037574.2	-1.41316	0.014813	DOWN	2.168444	0.000203	UP
NONMMUT124088.1	-1.42148	0.014843	DOWN	1.944136	0.000376	UP
NONMMUT057396.2	-1.89213	0.015096	DOWN	1.904552	0.00952	UP
ENSMUST00000133752	-1.46396	0.015098	DOWN	5.176146	1.65E-16	UP
NONMMUT096936.1	-3.56043	0.015133	DOWN	2.480165	0.040283	UP
ENSMUST00000141741	-1.18887	0.01529	DOWN	1.045485	0.017604	UP
MSTRG.59570.45	-5.72125	0.015675	DOWN	Inf	0.000128	UP

NONMMUT097275.1	-1.13625	0.015862	DOWN	1.514779	9.02E-05	UP
NONMMUT152961.1	-2.51886	0.015924	DOWN	2.213307	0.020143	UP
NONMMUT122272.1	#NAME?	0.015946	DOWN	Inf	0.015738	UP
NONMMUT117747.1	-1.50106	0.016157	DOWN	3.582312	1.91E-05	UP
NONMMUT053180.2	-1.40608	0.016305	DOWN	1.149974	0.021075	UP
ENSMUST00000148776	-2.7907	0.017089	DOWN	2.650018	0.021561	UP
NONMMUT027011.2	-1.15482	0.017101	DOWN	1.108921	0.034708	UP
NONMMUT019952.2	-1.10683	0.018104	DOWN	1.707268	5.94E-05	UP
NONMMUT092128.1	#NAME?	0.018265	DOWN	Inf	0.017434	UP
NONMMUT053809.2	-1.31157	0.018482	DOWN	1.949057	0.00464	UP
NONMMUT069789.2	-1.82379	0.018486	DOWN	2.725464	0.015079	UP
ENSMUST00000223775	#NAME?	0.019363	DOWN	Inf	0.019266	UP
NONMMUT024486.2	-2.45709	0.020067	DOWN	2.670171	0.010272	UP
ENSMUST00000222879	#NAME?	0.020107	DOWN	Inf	0.019076	UP
NONMMUT139602.1	#NAME?	0.020112	DOWN	Inf	0.01908	UP
NONMMUT088281.1	-1.48738	0.021063	DOWN	1.931729	0.002624	UP
ENSMUST00000200021	-2.08909	0.021772	DOWN	2.270351	0.015145	UP
NONMMUT071896.2	-1.63086	0.021936	DOWN	1.249926	0.041472	UP
NONMMUT033133.2	-1.16109	0.021966	DOWN	1.175563	0.025135	UP
NONMMUT025156.2	#NAME?	0.022443	DOWN	Inf	0.019017	UP
NONMMUT072803.2	-1.64588	0.022589	DOWN	3.376565	0.000105	UP
NONMMUT141001.1	#NAME?	0.023454	DOWN	Inf	0.023249	UP
NONMMUT108217.1	-1.59622	0.023566	DOWN	1.44856	0.021622	UP
MSTRG.4370.7	-4.6776	0.024086	DOWN	Inf	0.00795	UP
NONMMUT115209.1	-1.20073	0.024149	DOWN	Inf	5.56E-09	UP
NONMMUT058611.2	-1.22303	0.024169	DOWN	1.702397	0.001572	UP
NONMMUT115213.1	-1.01269	0.024829	DOWN	2.476503	2.06E-06	UP
NONMMUT051122.2	-1.54937	0.02496	DOWN	1.980156	0.00587	UP
NONMMUT070891.2	-1.0658	0.025157	DOWN	2.396573	1.29E-05	UP
NONMMUT026732.2	-1.76175	0.025196	DOWN	1.754716	0.018827	UP
NONMMUT080105.1	#NAME?	0.025834	DOWN	Inf	0.025031	UP
NONMMUT072383.2	#NAME?	0.025837	DOWN	Inf	0.025034	UP
ENSMUST00000148740	#NAME?	0.025838	DOWN	Inf	0.025036	UP
NONMMUT115226.1	-1.84304	0.02653	DOWN	1.476965	0.026453	UP
NONMMUT115225.1	-1.47418	0.026904	DOWN	2.363205	0.003433	UP
NONMMUT122614.1	-2.69109	0.027082	DOWN	1.967455	0.040933	UP
NONMMUT086017.1	-2.08968	0.0276	DOWN	2.847495	0.002474	UP
NONMMUT012225.2	-2.11837	0.027925	DOWN	2.041286	0.021686	UP
NONMMUT091178.1	#NAME?	0.028107	DOWN	Inf	0.022509	UP
NONMMUT013067.2	-1.6	0.028404	DOWN	1.138685	0.030262	UP
NONMMUT001628.2	-1.27144	0.028758	DOWN	1.446204	0.008075	UP
NONMMUT115260.1	-1.76944	0.029052	DOWN	1.55933	0.01743	UP
NONMMUT009151.2	-3.23479	0.029263	DOWN	Inf	0.000869	UP
NONMMUT115243.1	-1.43297	0.029386	DOWN	2.48319	0.000816	UP
NONMMUT133924.1	-3.06412	0.030135	DOWN	Inf	0.005792	UP
NONMMUT022944.2	-2.37062	0.030591	DOWN	2.866859	0.011084	UP
NONMMUT101552.1	#NAME?	0.031775	DOWN	Inf	0.031412	UP
NONMMUT091458.1	-1.86385	0.033648	DOWN	2.050796	0.014275	UP
NONMMUT151928.1	-1.00599	0.034844	DOWN	1.857114	0.001829	UP
NONMMUT115265.1	-2.72498	0.035522	DOWN	Inf	0.002431	UP

NONMMUT030183.2	#NAME?	0.035557	DOWN		Inf	0.034423	UP
NONMMUT050997.2	#NAME?	0.035557	DOWN		Inf	0.034429	UP
NONMMUT145722.1	-1.3091	0.035961	DOWN		1.422464	0.001891	UP
ENSMUST00000139288	-2.31306	0.036132	DOWN		Inf	0.001452	UP
NONMMUT151738.1	-1.19464	0.036304	DOWN		1.292011	0.013875	UP
NONMMUT140348.1	#NAME?	0.036884	DOWN		Inf	0.034806	UP
NONMMUT139186.1	-3.41217	0.037095	DOWN		Inf	0.00063	UP
NONMMUT114934.1	-1.12399	0.038027	DOWN		1.994975	0.000116	UP
NONMMUT113266.1	-2.0502	0.039618	DOWN		2.373116	0.018741	UP
ENSMUST00000132822	#NAME?	0.039875	DOWN		Inf	0.037187	UP
ENSMUST00000127284	-1.12785	0.041152	DOWN		1.625346	0.000105	UP
NONMMUT043749.2	#NAME?	0.041169	DOWN		Inf	0.041351	UP
NONMMUT013388.2	-2.88632	0.041706	DOWN		4.198178	0.005498	UP
NONMMUT013809.2	#NAME?	0.041885	DOWN		Inf	0.040987	UP
ENSMUST00000217446	-2.71213	0.042129	DOWN		Inf	0.004796	UP
NONMMUT106068.1	-1.82906	0.042269	DOWN		1.1998	0.017972	UP
NONMMUT076502.1	#NAME?	0.042487	DOWN		Inf	0.040355	UP
NONMMUT030215.2	#NAME?	0.042545	DOWN		Inf	0.041568	UP
NONMMUT153473.1	#NAME?	0.042607	DOWN		Inf	0.04032	UP
NONMMUT124745.1	-1.80603	0.042708	DOWN		1.788541	0.043498	UP
NONMMUT102916.1	-1.04486	0.043111	DOWN		1.022839	0.042276	UP
NONMMUT069496.2	-1.84807	0.043293	DOWN		4.834189	3.69E-24	UP
NONMMUT027240.2	#NAME?	0.04447	DOWN		Inf	0.044343	UP
NONMMUT083152.1	#NAME?	0.044501	DOWN		Inf	0.044315	UP
ENSMUST00000181077	-1.53888	0.04698	DOWN		1.860749	0.019802	UP
ENSMUST00000180830	-1.47821	0.047871	DOWN		1.811377	0.002511	UP
NONMMUT073684.2	-1.44185	0.048323	DOWN		3.80353	0.0002	UP
NONMMUT129985.1	#NAME?	0.048829	DOWN		Inf	0.048369	UP
ENSMUST00000152575	-1.26917	0.04905	DOWN		1.568925	0.013839	UP
NONMMUT046124.2	-1.99169	0.049254	DOWN		2.668716	0.011762	UP
NONMMUT093335.1	-1.01537	0.049281	DOWN		1.609595	0.001844	UP
NONMMUT074146.2	-1.93989	0.049314	DOWN		2.071069	0.037101	UP
ENSMUST00000188137	-1.20997	0.049968	DOWN		1.025745	0.048669	UP
