# Supplementary material for: Resveratrol Alleviates Skeletal Muscle Insulin Resistance by Downregulating Long Noncoding RNA
Source: Int J Endocrinol. 2022 Jan 19;2022:2539519. doi: 10.1155/2022/2539519 (PMC8791716; doi:10.1155/2022/2539519)
Supplement: Supplementary Materials — All differentially expressed lncRNAs and mRNAs in the control, HFD, and HFD + RSV groups are listed in Supplementary Tables S1 and S2. [file 2539519.f1.zip › 2539519.f1/Supplementary Table 2.pdf]

All_differentially_expressed_mRNAs_in_the__control__HFD__and_HFD+RSV_groups

gene name	log2FC	P-value	updown (RHvs HD)	log2FC	P-value	updown (HD vs ND)
Slc22a18	1.1337055	0.037414	UP	-1.37464	0.005434	DOWN
Drp2	1.7620168	6.18E-07	UP	-1.3602	0.000305	DOWN
Bcl6b	1.3381298	0.002695	UP	-1.39862	1.89E-10	DOWN
Pon1	1.5291377	0.000167	UP	-2.93332	4.96E-09	DOWN
Rab3b	3.2543477	0.000539	UP	-3.70665	0.000144	DOWN
Prlr	1.2833842	0.016403	UP	-1.70826	0.000926	DOWN
Dnase1	1.404254	0.016202	UP	-1.49264	0.00241	DOWN
Atp7b	1.7918932	0.044459	UP	-2.00586	0.027222	DOWN
Fzd3	1.1364598	0.010738	UP	-1.33576	0.000916	DOWN
Fhl2	1.3266949	0.003303	UP	-1.62672	0.000478	DOWN
Kif19a	1.5022578	0.001265	UP	-1.14672	0.006249	DOWN
Spire2	1.8411472	0.029013	UP	-1.94725	0.002649	DOWN
Bcas1	1.7179001	0.002295	UP	-1.04182	0.015404	DOWN
Myl2	2.2254932	0.001311	UP	-3.73321	7.15E-06	DOWN
Kif1a	1.9159254	0.000224	UP	-1.66421	2.61E-05	DOWN
Amdhd1	Inf	0.03783	UP	#NAME?	8.04E-05	DOWN
Tenm1	Inf	0.03634	UP	#NAME?	0.012257	DOWN
Tubb1	1.7869047	0.0287	UP	-1.90536	0.01363	DOWN
Wt1	Inf	0.02235	UP	#NAME?	0.022529	DOWN
Pmp22	1.2722106	0.007779	UP	-1.10369	0.02437	DOWN
Ddit4	1.3110335	2.84E-05	UP	-2.66711	1.58E-11	DOWN
Nsg2	2.743108	0.011766	UP	-2.32788	0.030427	DOWN
Cyfip2	1.2158008	0.019548	UP	-1.43267	0.001628	DOWN
Rasgef1c	1.4059895	0.013403	UP	-1.2224	0.036363	DOWN
Adcy1	1.6324421	0.001863	UP	-1.52901	0.000695	DOWN
Mboat2	1.2765518	0.007568	UP	-1.60971	0.002295	DOWN
Doc2b	1.4832763	0.001963	UP	-2.27032	7.59E-10	DOWN
Krt36	2.3213612	0.020524	UP	-2.6894	0.001998	DOWN
Fos	1.1269824	0.002405	UP	-1.07258	0.000206	DOWN
Degs2	1.7477845	0.044029	UP	-1.83473	0.037318	DOWN
Eci3	1.7474092	0.020357	UP	-3.88875	4.28E-10	DOWN
Drd1	1.1978423	0.03231	UP	-1.66244	0.014758	DOWN
Zfp346	1.6816061	2.63E-07	UP	-1.76233	2.40E-07	DOWN
Ocln	1.3393853	0.014038	UP	-1.48714	0.020228	DOWN
Elovl7	1.4125358	0.009919	UP	-1.24473	0.016851	DOWN
Hcn1	1.3884883	0.005171	UP	-1.42958	0.000444	DOWN
Dct	Inf	0.004746	UP	#NAME?	0.000772	DOWN
Cldn1	1.1601187	0.010503	UP	-1.67361	0.000177	DOWN
Clic6	1.7862734	0.009674	UP	-1.99517	0.007697	DOWN
Cxcl13	1.3716518	0.003807	UP	-2.58623	2.98E-05	DOWN
Lrrc71	1.0011775	0.048202	UP	-1.16508	0.023094	DOWN
Jakmip2	3.0474257	0.029555	UP	-2.62761	0.027009	DOWN
Gnal	1.2405875	0.012479	UP	-1.43949	0.002415	DOWN
Cidea	1.8523181	0.004372	UP	-6.49891	3.70E-19	DOWN
Ankrd1	1.9216575	0.042013	UP	-1.77744	0.020225	DOWN
Ctsw	Inf	0.021247	UP	#NAME?	7.02E-05	DOWN
Cdh9	2.6744135	0.000112	UP	-3.17518	0.000119	DOWN
Gli1	1.0551415	0.012189	UP	-1.53426	1.61E-05	DOWN
Cyp2e1	1.6748054	0.005926	UP	-4.30802	1.83E-16	DOWN
Il6	1.5105086	0.017765	UP	-1.52953	0.030867	DOWN
Sntg1	Inf	0.042907	UP	#NAME?	0.00275	DOWN

Tnni1	2.5513184	0.000608	UP	-3.74535	2.38E-05	DOWN
Nfasc	1.7411411	0.000576	UP	-1.39966	0.000303	DOWN
F5	1.3480548	0.012093	UP	-2.33839	3.04E-08	DOWN
Enkur	1.3529996	0.015587	UP	-1.29667	0.025201	DOWN
Cubn	1.776294	0.023347	UP	-2.18661	0.009666	DOWN
Nr4a2	1.600286	0.003942	UP	-1.56675	1.35E-06	DOWN
Galnt3	3.1236456	0.010879	UP	-2.47881	0.008086	DOWN
Hdc	1.3766361	0.004023	UP	-2.01409	2.57E-05	DOWN
Mal	2.0917632	0.00025	UP	-1.66489	0.000405	DOWN
Car3	1.0017694	0.002082	UP	-2.02138	2.35E-08	DOWN
Pex5l	1.746227	0.00208	UP	-1.34461	0.00512	DOWN
Wnt2b	1.5278842	0.008061	UP	-1.26686	0.007583	DOWN
Hmgcs2	1.1427217	0.003521	UP	-1.40982	4.75E-05	DOWN
Tpm3	1.0251486	0.000917	UP	-1.95099	3.35E-06	DOWN
Myoz2	1.2017412	0.013	UP	-2.15563	0.000115	DOWN
Gabrr1	1.0042139	0.027277	UP	-1.12133	0.007296	DOWN
Fmn2	1.5888366	0.01072	UP	-1.4541	0.001734	DOWN
Mfsd2a	2.1757297	0.000638	UP	-4.11817	4.69E-06	DOWN
Plk3	1.4682272	0.003694	UP	-1.83355	2.44E-07	DOWN
Stmn1	1.4599176	0.004151	UP	-1.55255	2.97E-05	DOWN
Slc30a2	1.6991836	6.07E-06	UP	-1.68498	1.63E-06	DOWN
Fosl2	1.0233397	4.21E-05	UP	-1.16247	1.85E-08	DOWN
Cgref1	1.8584718	0.015316	UP	-1.98391	0.002235	DOWN
Spp1	2.4650167	2.88E-05	UP	-2.30117	1.20E-06	DOWN
Asphd2	1.0106031	0.025998	UP	-1.04528	0.020154	DOWN
Atp2a2	1.111872	0.003862	UP	-2.18998	5.53E-06	DOWN
Cd27	1.0467932	0.020947	UP	-1.81532	4.40E-05	DOWN
Csrp3	1.5256331	0.001633	UP	-2.58764	0.000202	DOWN
Acsm3	1.5849314	0.022282	UP	-4.884	2.86E-15	DOWN
Acsm5	1.0983429	0.044028	UP	-2.1189	1.78E-05	DOWN
Bmx	1.2739234	0.005274	UP	-2.10697	5.95E-05	DOWN
Tnfsf13b	1.1422045	0.010129	UP	-1.20579	0.016517	DOWN
Gpm6a	1.2501289	0.006217	UP	-1.68356	0.000313	DOWN
Slit2	1.7643144	0.004194	UP	-1.39341	0.006878	DOWN
Ucp1	2.2881961	0.012901	UP	-8.01324	9.08E-11	DOWN
Irx5	2.8428799	0.009867	UP	-2.63138	0.000432	DOWN
Mt3	1.2911768	0.005362	UP	-1.22738	0.007211	DOWN
Pllp	2.2713895	0.000187	UP	-2.09093	4.71E-07	DOWN
Barx2	1.4650504	0.003007	UP	-1.45041	0.007598	DOWN
Mpzl2	1.1184088	0.000765	UP	-4.00237	8.47E-23	DOWN
Tinag	3.9415974	0.00321	UP	-3.42985	0.004488	DOWN
Arpp21	1.0154819	0.007971	UP	-1.65801	7.05E-05	DOWN
Ugt8a	2.5773213	0.00015	UP	-1.94324	0.00014	DOWN
Sox10	1.2774666	0.008898	UP	-1.00199	0.004996	DOWN
S100b	1.5861953	0.000709	UP	-2.53907	0.000151	DOWN
Fa2h	1.2992428	0.029667	UP	-1.18303	0.034451	DOWN
Egr3	1.870639	0.008758	UP	-2.57632	1.35E-07	DOWN
Kcnk1	1.4772428	0.007284	UP	-1.28157	0.004708	DOWN
Pla2g3	1.9206611	0.011095	UP	-1.83338	0.038519	DOWN
Shc4	1.8627443	1.36E-08	UP	-1.31095	0.003579	DOWN
Ccr3	3.2624139	0.009446	UP	-4.08782	0.000509	DOWN
Sostdc1	1.9160845	0.019805	UP	-2.52032	0.000466	DOWN
Slc18a1	1.5628681	0.009195	UP	-2.1745	0.000183	DOWN
Mag	1.6147615	0.013186	UP	-1.57245	0.000186	DOWN

Igflr1	Inf	0.010242	UP	#NAME?	0.018618	DOWN
Myom3	1.342974	0.002056	UP	-2.16844	1.06E-05	DOWN
Serpine1	1.2426234	0.00101	UP	-2.09645	5.56E-05	DOWN
Fbxo34	1.2032436	0.006527	UP	-1.69026	0.000875	DOWN
Acer2	1.0537219	0.003407	UP	-2.31605	6.54E-08	DOWN
Kcna6	1.1506297	0.011805	UP	-1.11915	0.01092	DOWN
Egr1	1.7580684	0.000108	UP	-1.71121	5.02E-05	DOWN
Elovl3	3.024231	0.025756	UP	-9.01543	1.29E-10	DOWN
3930402G23R	1.9065843	0.015447	UP	-2.66495	0.000183	DOWN
Cfap157	Inf	0.03056	UP	#NAME?	0.039925	DOWN
Ak5	1.5752571	0.018229	UP	-1.67744	0.00086	DOWN
Nrn1	1.5640406	0.005661	UP	-2.02911	2.83E-05	DOWN
Svs1	4.1769717	0.037941	UP	-5.29519	0.003726	DOWN
Zfp750	2.6251097	0.00087	UP	-1.91117	0.006574	DOWN
Lyplal1	1.0767008	0.010495	UP	-2.38031	1.13E-08	DOWN
Pak7	Inf	0.013639	UP	#NAME?	0.026294	DOWN
Ptger4	1.4424748	0.001803	UP	-1.66732	2.05E-07	DOWN
Svs2	Inf	0.001283	UP	#NAME?	4.58E-05	DOWN
Cacng5	Inf	0.02162	UP	#NAME?	0.007833	DOWN
Plekha4	1.3066692	0.002928	UP	-1.11677	0.00307	DOWN
Spns2	1.1123944	2.78E-05	UP	-1.40598	2.90E-11	DOWN
Rimklb	2.5487696	0.002301	UP	-1.97961	0.002963	DOWN
Slco4c1	Inf	0.021681	UP	#NAME?	0.034864	DOWN
Myh6	1.3710079	0.040009	UP	-2.33474	0.0002	DOWN
Elovl6	1.1608389	0.002816	UP	-6.25882	2.26E-19	DOWN
Tox	1.4142921	0.001254	UP	-1.21833	0.015176	DOWN
Sdk2	1.5878589	9.29E-05	UP	-2.39957	1.13E-06	DOWN
Mbp	1.3164083	0.000193	UP	-1.53898	4.24E-07	DOWN
Gck	1.0621549	0.008011	UP	-1.66141	4.83E-06	DOWN
Mgat3	1.0382849	0.007315	UP	-1.02613	0.002141	DOWN
Mustn1	1.6613401	0.02006	UP	-1.66164	0.002112	DOWN
Ttc9	1.3018418	0.000413	UP	-1.80348	0.000361	DOWN
Smco3	2.3293118	0.000198	UP	-1.71384	0.008468	DOWN
Ubxn10	2.0526041	0.014275	UP	-2.54672	0.001423	DOWN
Ncmap	1.5741079	0.00278	UP	-1.49585	0.001078	DOWN
P2ry4	Inf	0.008886	UP	#NAME?	0.038265	DOWN
Slc15a5	2.2856871	0.000414	UP	-1.58975	0.00048	DOWN
Prrt2	2.4833767	0.005936	UP	-2.00671	0.007392	DOWN
Proser2	1.1743558	0.00494	UP	-1.48515	0.000552	DOWN
Gldn	1.1197049	0.011748	UP	-1.07534	0.029748	DOWN
Hepacam	2.5785476	0.002221	UP	-2.2947	0.00489	DOWN
Fam178b	1.9266133	0.003232	UP	-1.595	0.003832	DOWN
Kctd4	Inf	0.008516	UP	#NAME?	0.004221	DOWN
Bdh1	1.0715635	0.039647	UP	-2.0466	0.000483	DOWN
Vat1l	1.578749	0.005636	UP	-1.8374	0.000576	DOWN
Cdh19	1.066028	0.00294	UP	-1.44895	0.000102	DOWN
Ankfn1	2.5111533	0.002502	UP	-1.70924	0.005164	DOWN
Pygo2	1.4556088	0.000167	UP	-1.28536	0.017698	DOWN
Tmem72	2.8208095	0.011039	UP	-3.04876	0.0042	DOWN
Cfap100	2.2296329	0.001055	UP	-1.63319	0.017507	DOWN
Frmd3	1.9994911	0.000542	UP	-1.66899	0.003509	DOWN
Tenm2	1.0579895	0.010664	UP	-1.27034	0.00505	DOWN
Rasd1	1.8553751	0.001176	UP	-3.8766	2.12E-15	DOWN
Fhl4	1.1217669	0.045133	UP	-1.1723	0.034471	DOWN

Vwc2	Inf	0.005033	UP	#NAME?	0.00298	DOWN
Igfn1	1.1172996	0.007578	UP	-1.46442	0.013074	DOWN
Pmp2	4.5278817	7.96E-05	UP	-4.44972	7.58E-05	DOWN
Lrrtm4	Inf	0.03421	UP	#NAME?	0.016326	DOWN
Junb	1.0966674	0.001262	UP	-1.44993	8.26E-07	DOWN
Cyp2f2	1.2741672	0.032209	UP	-3.61353	3.52E-12	DOWN
Sv2b	1.8354298	0.000481	UP	-3.0898	1.51E-05	DOWN
Myh7	3.1961409	0.000961	UP	-4.66692	2.39E-05	DOWN
Ptprt	1.2952518	0.043469	UP	-1.80358	0.003119	DOWN
Prx	1.9030756	0.000307	UP	-1.52763	0.000283	DOWN
Adamts18	1.3100541	0.039405	UP	-1.50499	0.004408	DOWN
Cadm4	1.6676487	0.002179	UP	-1.41439	0.001238	DOWN
Kcnmb4	2.1231609	0.042754	UP	-2.46683	0.001887	DOWN
Lmntd1	3.5167796	0.000955	UP	-3.00979	0.002232	DOWN
Cndp1	Inf	0.008532	UP	#NAME?	0.026412	DOWN
Mpz	2.4235933	6.54E-05	UP	-1.92778	1.18E-05	DOWN
Gsta2	3.9922032	0.004633	UP	-3.96921	0.036122	DOWN
Capn11	3.4310473	9.77E-06	UP	-4.32608	1.21E-08	DOWN
Nap1l1	1.2932503	0.000581	UP	-1.66682	2.85E-06	DOWN
Ddn	2.2745782	0.014198	UP	-1.79668	0.003874	DOWN
Myl3	2.3811431	0.008413	UP	-4.11167	8.91E-05	DOWN
Rufy4	1.762066	0.027984	UP	-3.81182	1.39E-08	DOWN
Ces2c	2.7003762	0.003073	UP	-3.50224	2.11E-06	DOWN
Ifnlr1	Inf	0.042537	UP	#NAME?	0.012485	DOWN
Kcnj15	1.1303851	0.022233	UP	-2.05039	3.37E-05	DOWN
Sim2	1.6755062	0.005489	UP	-1.76258	0.000123	DOWN
Msln	1.4031233	0.00599	UP	-1.6891	0.017633	DOWN
Tnfrsf11b	1.3813213	0.032043	UP	-1.37629	0.038828	DOWN
Tnnt1	2.4959276	0.000538	UP	-3.64057	5.48E-06	DOWN
Aox3	1.0296063	0.028206	UP	-1.42769	0.001404	DOWN
Cldn19	1.7067056	0.002505	UP	-1.56374	0.000289	DOWN
Lgi1	2.0315908	0.001291	UP	-2.11908	2.83E-05	DOWN
Il2rb	1.6036858	0.020691	UP	-2.19772	2.41E-05	DOWN
Mmp27	1.1913815	0.04508	UP	-1.27782	0.027736	DOWN
Gpr179	3.5693902	0.009783	UP	-3.67922	0.015575	DOWN
Ppia	1.1192907	2.62E-05	UP	-1.19672	0.001831	DOWN
Arhgdig	1.5882065	0.004403	UP	-1.51033	0.01744	DOWN
Mob3b	1.0238104	0.003305	UP	-1.47925	2.31E-06	DOWN
Krtdap	1.8697773	0.009492	UP	-1.89494	0.007156	DOWN
Gm10658	1.2921501	0.039646	UP	-1.47978	0.044266	DOWN
Gm826	1.0109303	0.007546	UP	-1.38869	0.005256	DOWN
Ube2n	1.3227921	0.008027	UP	-1.50551	0.002826	DOWN
AU019990	1.576893	0.018818	UP	-1.82796	0.004538	DOWN
Gas2l3	1.1390464	0.006303	UP	-1.02807	0.020453	DOWN
Sptbn5	1.2740002	0.003898	UP	-1.10708	0.002407	DOWN
Iglc2	Inf	0.012601	UP	#NAME?	0.030258	DOWN
Gm8818	3.2555113	0.006078	UP	-3.17352	0.002513	DOWN
Cntf	1.4527674	0.003031	UP	-1.33808	0.014886	DOWN
Stard6	1.4523281	0.042521	UP	-1.64199	0.022625	DOWN
Gm5847	Inf	0.035947	UP	#NAME?	0.000463	DOWN
Gm12240	1.3488969	0.022966	UP	-1.59548	0.012511	DOWN
Mir1904	1.1113314	0.011967	UP	-1.12175	0.00477	DOWN
Gm830	1.486829	0.005824	UP	-1.70465	0.003748	DOWN
Gm12295	Inf	0.035751	UP	#NAME?	1.76E-05	DOWN

B430219N15R	1.7470109	0.019223	UP	-2.91797	1.55E-05	DOWN
Sbk3	1.3573345	0.022405	UP	-2.5462	1.72E-05	DOWN
Hotair	1.8655187	0.04719	UP	-1.7384	0.006007	DOWN
Sap30bpos	2.6762014	0.024012	UP	-2.93371	0.007987	DOWN
Gm43213	1.7315222	0.026208	UP	-2.45654	2.86E-06	DOWN
Gm24530	1.5160479	0.038047	UP	-1.96389	0.020701	DOWN
Usp49	1.4051651	0.00151	UP	-1.67627	2.02E-05	DOWN
Gm17034	1.3596921	0.038274	UP	-1.51507	0.033745	DOWN
Gm20425	Inf	0.004626	UP	#NAME?	5.47E-11	DOWN
Apold1	1.5839344	0.000875	UP	-1.98231	6.16E-16	DOWN
Apol11b	2.1143183	0.000579	UP	-1.76145	0.012619	DOWN
Tnnc1	2.9425546	0.000595	UP	-4.14904	5.24E-06	DOWN
Gm20427	1.4269699	0.033604	UP	-2.85579	1.58E-05	DOWN
Gm20656	Inf	0.00307	UP	#NAME?	0.00143	DOWN
Igha	Inf	7.45E-05	UP	#NAME?	0.000404	DOWN
Lhx8	3.9938547	0.007842	UP	-5.54154	1.26E-05	DOWN
Gm26546	2.4521787	0.019434	UP	-2.30657	0.029807	DOWN
4930539J05R	1.2111437	0.046218	UP	-1.73364	0.001893	DOWN
Gm26510	1.2198864	0.020304	UP	-1.33533	0.011652	DOWN
4732419C18R	2.8996587	0.045999	UP	-5.49776	1.00E-07	DOWN
Gm26751	2.584699	0.031104	UP	-2.79783	0.018081	DOWN
Mhrt	3.9551746	0.001717	UP	-5.23302	1.18E-06	DOWN
9530059O14R	2.5012503	0.000178	UP	-1.68389	0.000514	DOWN
Gm6225	1.3932157	0.04718	UP	-1.57809	0.021859	DOWN
4933405D12R	Inf	0.018623	UP	#NAME?	0.015655	DOWN
Gm26679	2.2060704	0.029847	UP	-2.45561	0.03242	DOWN
Gm18303	Inf	0.003411	UP	#NAME?	0.007119	DOWN
1700030C10R	1.8866227	0.008786	UP	-2.18363	0.014636	DOWN
Gm28874	1.493199	0.012722	UP	-1.95788	0.004319	DOWN
Gm17971	1.0824731	0.012236	UP	-1.01911	0.001132	DOWN
1810041H14R	1.7260643	0.001858	UP	-2.06661	0.000937	DOWN
Gm37541	1.0850032	0.045496	UP	-1.37185	0.011088	DOWN
Gm37783	1.0368967	0.008105	UP	-1.33174	1.82E-06	DOWN
Gm38036	1.1678329	0.001111	UP	-1.46072	2.05E-05	DOWN
Gm38357	1.1515923	1.18E-05	UP	-1.58082	3.13E-07	DOWN
Gm37537	1.0136308	0.014094	UP	-1.05529	0.004648	DOWN
Gm34302	Inf	0.012449	UP	#NAME?	0.008521	DOWN
2900035J10R	1.1942947	0.04724	UP	-1.77521	0.004196	DOWN
Gm37510	1.0301203	0.008302	UP	-1.19184	0.004453	DOWN
Gm43314	1.9551895	0.006671	UP	-2.0633	0.002978	DOWN
Gm43797	1.000953	0.003681	UP	-1.70083	6.88E-07	DOWN
Gm42946	1.2621475	0.012031	UP	-1.97232	0.000143	DOWN
Gm43503	1.0775432	0.012298	UP	-1.53561	0.000367	DOWN
Gm45234	2.1125241	0.018578	UP	-2.28123	0.009898	DOWN
Gm44440	Inf	0.001052	UP	#NAME?	0.011902	DOWN
Gm45203	1.0756433	0.011794	UP	-1.96155	1.39E-06	DOWN
Gm49320	7.9556429	0.000637	UP	-9.05838	5.85E-08	DOWN
AI314278	Inf	0.003111	UP	#NAME?	8.46E-07	DOWN
Gm36356	2.2536317	0.038361	UP	-2.11302	0.025706	DOWN
Gm45424	1.9148886	0.007532	UP	-2.20112	0.007366	DOWN
Gm48114	1.6393039	0.000508	UP	-1.42285	0.001811	DOWN
Gm49380	3.014319	2.49E-07	UP	-1.57209	0.043509	DOWN
Gm9129	Inf	0.031055	UP	#NAME?	0.013263	DOWN
Gm47593	5.3523367	0.000714	UP	-6.18561	1.58E-06	DOWN

Gm40617	2.4270927	0.019626	UP	-3.61837	0.000182	DOWN
Gm33111	Inf	0.036025	UP	#NAME?	0.00686	DOWN
Gm5427	3.0500316	0.012196	UP	-2.85517	0.019714	DOWN
Gm18042	2.1838293	0.020846	UP	-1.97768	0.043499	DOWN
Gm48302	1.2227674	0.00137	UP	-1.82305	3.57E-10	DOWN
Gm48593	1.4959145	0.000502	UP	-1.43475	1.84E-07	DOWN
Gm47920	1.0220116	0.001157	UP	-1.40999	1.59E-07	DOWN
Gm49311	1.452445	0.003503	UP	-1.52245	0.007642	DOWN
Gm46516	Inf	0.031388	UP	#NAME?	0.0163	DOWN
C230086J09R	1.3312718	0.004005	UP	-1.3466	0.006132	DOWN
Gm31251	2.2946895	0.048991	UP	-3.67794	5.29E-05	DOWN
Gm30970	1.0295415	0.045753	UP	-2.01769	0.000141	DOWN
Gm2682	Inf	0.001709	UP	#NAME?	1.11E-05	DOWN
Elf3	-1.121181	0.026797	DOWN	1.339695	0.002555	UP
Pax3	-1.094223	0.02664	DOWN	1.163196	2.79E-05	UP
Ccl8	-2.426228	0.003003	DOWN	1.470391	0.034412	UP
Pcsk1	-1.141435	0.001394	DOWN	1.960889	6.87E-07	UP
Nr4a3	-1.5554	3.47E-05	DOWN	1.225581	0.000182	UP
Sh3gl2	-2.081071	5.64E-06	DOWN	2.803522	1.83E-09	UP
Ppm1n	-1.058253	0.012598	DOWN	3.000682	2.93E-08	UP
Dkk3	-1.518873	0.000588	DOWN	2.060882	1.16E-07	UP
Zfp92	-1.553055	0.027863	DOWN	1.216859	0.035556	UP
Pou2f3	-2.672267	0.010465	DOWN	2.046599	0.032114	UP
Cish	-2.487363	1.64E-20	DOWN	2.1587	1.43E-14	UP
Vax2	-3.73425	0.002507	DOWN	1.550073	0.048685	UP
Ccl12	-3.965079	0.00141	DOWN	2.641596	0.0058	UP
Ccl7	-1.924166	0.005174	DOWN	1.669165	0.006831	UP
Megf11	-1.264218	0.028085	DOWN	1.597213	0.002069	UP
Socs1	-2.361433	1.61E-06	DOWN	2.44948	4.05E-07	UP
4932438H23R	-1.872502	0.019401	DOWN	1.740581	0.019357	UP
Cd209e	-1.520768	0.033734	DOWN	2.146486	0.001096	UP
Map6d1	-1.726512	0.047048	DOWN	3.074208	0.007729	UP
Rell2	-1.42277	0.019966	DOWN	1.235684	0.039337	UP
Apol7c	#NAME?	0.001161	DOWN	3.583676	0.005716	UP
Mmp12	#NAME?	1.16E-05	DOWN	4.239946	0.000463	UP
C130026L21R	-2.523226	0.024031	DOWN	2.31491	0.02371	UP
Socs3	-1.461851	4.61E-05	DOWN	1.322875	2.70E-05	UP
Cldn23	#NAME?	0.001691	DOWN	2.491168	0.028549	UP
Rpl21-ps12	-1.839607	0.012602	DOWN	2.537707	0.000902	UP
Kcnh7	-1.111919	0.016375	DOWN	2.453351	3.86E-11	UP
Clca4a	-2.146688	0.002907	DOWN	2.729295	0.000758	UP
Kcng1	-1.700116	0.004115	DOWN	1.401149	0.003396	UP
Gm10782	#NAME?	0.01485	DOWN	Inf	0.013917	UP
Ighg2b	#NAME?	0.008172	DOWN	Inf	0.007726	UP
Utp14b	-1.280461	1.46E-09	DOWN	1.255252	3.33E-09	UP
Gm12856	#NAME?	0.001357	DOWN	2.047901	0.030731	UP
B230312C02R	-1.85503	2.38E-10	DOWN	1.846494	1.97E-10	UP
Gm15478	-1.167177	0.029683	DOWN	2.466703	1.04E-05	UP
Vax2os	-1.527861	0.03291	DOWN	1.537489	0.01731	UP
Gm16933	-1.186897	0.014228	DOWN	1.041971	0.020187	UP
4930512H18R	-1.208679	0.003987	DOWN	3.787461	3.47E-18	UP
Gm12199	-1.032603	0.004768	DOWN	1.507311	9.17E-05	UP
D430001F17R	#NAME?	0.008152	DOWN	2.862426	0.042373	UP
Gm15838	-3.236679	9.37E-06	DOWN	Inf	9.43E-10	UP

Gm16534	-1.020263	0.00184	DOWN	1.13025	0.000428	UP
Gm26876	-3.113808	5.15E-07	DOWN	3.811306	7.03E-09	UP
Gm26635	-1.535233	0.040218	DOWN	1.857674	0.020349	UP
Gm5253	#NAME?	0.03077	DOWN	Inf	0.029311	UP
Gm18342	#NAME?	0.043853	DOWN	Inf	0.042123	UP
Gm49347	-2.793878	0.016914	DOWN	2.69398	0.016677	UP
AY036118	-2.083637	0.022577	DOWN	2.26368	0.014427	UP
Gm9402	-2.956266	0.046539	DOWN	2.633552	0.044306	UP
Gm38534	#NAME?	3.73E-05	DOWN	1.808615	0.012789	UP
Gm49388	-2.530209	0.044372	DOWN	6.06138	1.92E-10	UP
Gm33543	-1.305677	0.029604	DOWN	2.661545	2.23E-13	UP
Gm47416	-2.70776	0.041958	DOWN	Inf	0.003844	UP
Gm48719	-1.435416	0.008835	DOWN	2.618363	1.87E-05	UP
Gm33958	#NAME?	0.017721	DOWN	Inf	0.017255	UP
Gm33489	-1.522658	0.024864	DOWN	1.206656	0.042682	UP
Gm47603	-1.361788	0.007302	DOWN	2.39615	2.51E-05	UP
Gm29676	-3.861329	0.002862	DOWN	3.283294	0.005301	UP
